# Cal‘MAM’ity at the Endoplasmic Reticulum-Mitochondrial Interface: A Potential Therapeutic Target for Neurodegeneration and Human Immunodeficiency Virus-Associated Neurocognitive Disorders

**DOI:** 10.3389/fnins.2021.715945

**Published:** 2021-10-21

**Authors:** Jessica Proulx, In-Woo Park, Kathleen Borgmann

**Affiliations:** Department of Microbiology, Immunology and Genetics, University of North Texas Health Science Center (HSC), Fort Worth, TX, United States

**Keywords:** mitochondria-associated ER membranes, Unfolded protein response, ER stress, calcium dysregulation, mitochondrial dysfunction, neuropathology, astrocytes

## Abstract

The endoplasmic reticulum (ER) is a multifunctional organelle and serves as the primary site for intracellular calcium storage, lipid biogenesis, protein synthesis, and quality control. Mitochondria are responsible for producing the majority of cellular energy required for cell survival and function and are integral for many metabolic and signaling processes. Mitochondria-associated ER membranes (MAMs) are direct contact sites between the ER and mitochondria that serve as platforms to coordinate fundamental cellular processes such as mitochondrial dynamics and bioenergetics, calcium and lipid homeostasis, autophagy, apoptosis, inflammation, and intracellular stress responses. Given the importance of MAM-mediated mechanisms in regulating cellular fate and function, MAMs are now known as key molecular and cellular hubs underlying disease pathology. Notably, neurons are uniquely susceptible to mitochondrial dysfunction and intracellular stress, which highlights the importance of MAMs as potential targets to manipulate MAM-associated mechanisms. However, whether altered MAM communication and connectivity are causative agents or compensatory mechanisms in disease development and progression remains elusive. Regardless, exploration is warranted to determine if MAMs are therapeutically targetable to combat neurodegeneration. Here, we review key MAM interactions and proteins both *in vitro* and *in vivo* models of Alzheimer’s disease, Parkinson’s disease, and amyotrophic lateral sclerosis. We further discuss implications of MAMs in HIV-associated neurocognitive disorders (HAND), as MAMs have not yet been explored in this neuropathology. These perspectives specifically focus on mitochondrial dysfunction, calcium dysregulation and ER stress as notable MAM-mediated mechanisms underlying HAND pathology. Finally, we discuss potential targets to manipulate MAM function as a therapeutic intervention against neurodegeneration. Future investigations are warranted to better understand the interplay and therapeutic application of MAMs in glial dysfunction and neurotoxicity.

## Introduction

Crosstalk amongst subcellular organelles is an intricate and essential phenomenon for coordinating intracellular communication and ultimately regulating cellular fate. In fact, organelles such as the nucleus, endoplasmic reticulum (ER), golgi apparatus, plasma membrane, mitochondria, lysosomes, peroxisomes and endosomes are now known to both functionally and physically interact with each other to carry out distinct cellular functions (Khan et al., 2019; Liao et al., 2020; Picca et al., 2020). Notably, this review will focus on the direct contact sites between the ER and mitochondria. The ER has many pivotal functions that regulate cellular function and physiology, including calcium (Ca^2+^) storage and release, lipid biogenesis, and protein folding, assembly, modification and sorting. Moreover, the ER is an intracellular stress sensor, which uses well-established quality control mechanisms such as the unfolded protein response (UPR) and ER-associated degradation (ERAD) signaling pathways to respond to cellular stress and maintain homeostasis (Vincenz-Donnelly and Hipp, 2017). Mitochondria, having often been renowned as the ‘powerhouse of the cell,’ are essential for ATP production, Ca^2+^ buffering as well as regulating various elements of cellular fate through metabolic, apoptotic, and redox signaling (Brand et al., 2013; Friedman and Nunnari, 2014). The direct contact sites between the ER and mitochondria are crucial to regulate both the dynamic structure and function of these two organelles (van Vliet et al., 2014).

## Mitochondria-Associated Endoplasmic Reticulum Membranes

**FIGURE 1:**
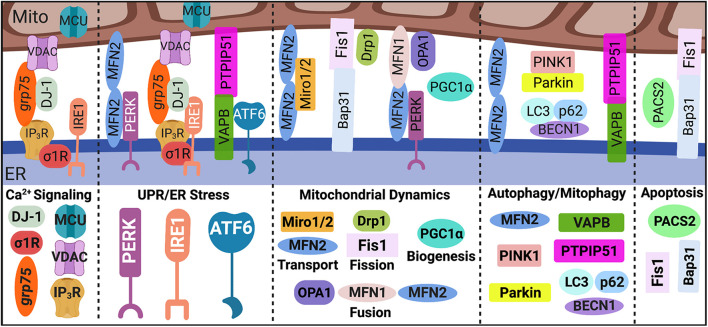
MAM proteome. Direct contact sites between the ER and mitochondria, MAMs, are tightly regulated by key tethering factors: MFN2, VAPB-PTPIP51, IP_3_R-grp75-DJ-1-VDAC, and Bap31-Fis1. Mediators within the ER-mitochondrial interface regulate distinct MAM-mediated mechanisms such as Ca^2+^ signaling, UPR/ER stress, mitochondrial dynamics, autophagy/mitophagy, and apoptosis. MAM, mitochondria-associated ER membrane; Mito, mitochondria; ER, endoplasmic reticulum; Ca^2+^, calcium; IP_3_R, inositol 1,4,5-triphosphate receptors; VDAC, voltage-dependent anion-selective channel; grp75, glucose-regulated protein 75 kDa; MCU, mitochondrial Ca^2+^ uniporter; σ1R, sigma-1 receptor; UPR, unfolded protein response; PERK, protein kinase RNA-like endoplasmic reticulum kinase; IRE1α, inositol-requiring protein 1α; ATF6, activating transcription factor 6; MFN, mitofusin; Miro, mitochondrial Rho GTPases; Drp1, dynamin-related protein 1; Fis1, fission 1; OPA1, optic atrophy protein 1; PGC1α, proliferator-activated receptor γ coactivator 1α; PINK1, phosphatase and tensin homolog-induced putative kinase; BECN1, beclin 1; LC3, microtubule-associated protein 1A/1B-light chain 3; VAPB, membrane protein-associated protein B; PTPIP51, protein tyrosine phosphatase-interacting protein 51; PACS2, phosphofurin acidic cluster sorting 2; and Bap31, B cell receptor-associated protein 31. Image created with BioRender.com.

While the first indication of direct ER-mitochondria contact was described in 1956, it took nearly four decades of continued exploration before the concept of a physical ER-mitochondrial interaction gained acceptance and the term, mitochondria-associated ER membranes (MAMs), was coined (Herrera-Cruz and Simmen, 2017). Following acceptance of this phenomenon, it was not until over 20 years later that we were able to produce the first comprehensive analysis of the MAM proteome (Herrera-Cruz and Simmen, 2017; Janikiewicz et al., 2018; Moltedo et al., 2019). Since these initial investigations, multiple mediators have been identified in regulating both the structure and function of the ER-mitochondrial interface, which are illustrated in Figure 1.

Strides have been made in our understanding of how MAMs are integral signaling platforms that regulate multiple cellular functions and maintain homeostasis. In addition to regulating the function and dynamics of both the ER and mitochondria, as discussed below, MAMs are considered central hubs for regulating key cellular processes including apoptosis, autophagy, Ca^2+^ and lipid homeostasis, inflammation, and inflammasome formation (Paillusson et al., 2016; Janikiewicz et al., 2018; Moltedo et al., 2019). Tether proteins between the ER and mitochondria serve as essential scaffolds in regulating MAM-mediated mechanisms whereas communication between these two organelles is primarily facilitated by Ca^2+^ and redox signaling (Marchi et al., 2017; Yoboue et al., 2018; Moltedo et al., 2019).

Briefly, the transfer of Ca^2+^ from the ER to mitochondria is facilitated by inositol 1,4,5-triphosphate receptors (IP_3_R) on the ER membrane, voltage-dependent anion-selective channel (VDAC) on the outer mitochondrial membrane (OMM), and cytosolic glucose-regulated protein 75 kDa (grp75), which stabilizes IP_3_R and VDAC association within the MAM interface. Notably, cytosolic DJ-1 is newly identified as a critical component in the IP_3_R-grp75-VDAC complex (Liu et al., 2019; Basso et al., 2020). On the inner mitochondrial membrane (IMM) mitochondrial Ca^2+^ uniporter (MCU) enables the Ca^2+^ transfer into the matrix, which increases the electrochemical potential and thus oxidative phosphorylation power (Rizzuto et al., 2012; van Vliet et al., 2014; Giorgi et al., 2018). On the ER membrane side of the interface, the sigma-1 receptor (σ1R) promotes of Ca^2+^ transfer by associating with IP_3_R (Hayashi and Su, 2007).

One of the most researched proteins involved in MAM tethering is mitofusin (MFN) 2, which localizes to both the OMM and ER membrane forming a homodimer as well as a heterodimer with MFN1 (de Brito and Scorrano, 2008; Leal and Martins, 2021). However, whether MFN2 positively or negatively regulates MAM tethering remains controversial across differing conditions and cell types (de Brito and Scorrano, 2008; Filadi et al., 2015; Janikiewicz et al., 2018; Moltedo et al., 2019; Leal and Martins, 2021); thus, functional outcomes of MFN2 manipulation can vary. Additional regulators of MAM tethering are vesicle-associated membrane protein-associated protein B (VAPB) on the ER membrane and protein tyrosine phosphatase-interacting protein 51 (PTPIP51) on the OMM (De Vos et al., 2012; Leal and Martins, 2021). Interestingly, MAMs are origin sites for autophagy initiation and autophagosome formation (Yang et al., 2020), which is negatively regulated by VAPB-PTPIP51 tethering (Gomez-Suaga et al., 2017). Finally, MAM tethering *via* ER-associated B cell receptor-associated protein 31 (Bap31) and mitochondrial fission 1 (Fis1) construct a scaffold for apoptotic signaling (Iwasawa et al., 2011), where phosphofurin acidic cluster sorting 2 (PACS2) is a pivotal regulator (Simmen et al., 2005).

Mitochondria-associated ER membranes are indispensable for ER physiology and health and are intricately involved in cellular responses to UPR/ER stress signaling. Interestingly, many studies have revealed distinct associations between the UPR sensors and MAM regulation both in response to and in the absence of ER stress, highlighting possible non-canonical functions for these proteins (Bravo et al., 2011; van Vliet et al., 2014; Saito and Imaizumi, 2018; van Vliet and Agostinis, 2018). Briefly, there are three arms to the UPR cascade: protein kinase RNA-like endoplasmic reticulum kinase (PERK), inositol-requiring protein 1α (IRE1α), and activating transcription factor 6 (ATF6). PERK is proposed as a key regulator of MAM tethering through direct interaction with MFN2 and is also linked to regulating mitochondrial dynamics and bioenergetics (Verfaillie et al., 2012; Munoz et al., 2013; Rainbolt et al., 2014; van Vliet and Agostinis, 2016; Lebeau et al., 2018; Balsa et al., 2019). IRE1α, which is commonly associated with cellular responses to infections or inflammation, is implicated in ER-mitochondrial Ca^2+^ transfer, mitochondrial respiration and redox homeostasis through associations with IP_3_R (Son et al., 2014; Carreras-Sureda et al., 2019) and/or σ1R (Mori et al., 2013). ATF6 is shown to both interact with and be regulated by the key MAM tethering protein VAPB (Gkogkas et al., 2008). Moreover, ATF6 can regulate lipid biosynthesis and ER expansion suggesting a possible interplay in MAM-mediated lipid homeostasis and ER-mitochondrial physiology (Bommiasamy et al., 2009). Most recently, ATF6 has also been implicated in ER-mitochondrial Ca^2+^ homeostasis (Burkewitz et al., 2020).

The ER and mitochondria contact sites are fundamental for regulating mitochondrial function, dynamics, and homeostasis. For example, Ca^2+^ and redox signaling between the ER and mitochondria are essential for regulating mitochondrial integrity and bioenergetic activity. Moreover, mitochondrial dynamics are regulated by a balance between fission and fusion both of which are coordinated by the ER-mitochondrial interface. In fact, fission requires the physical maneuvering of the ER membrane to constrict around the mitochondrion fission site which then recruits the primary regulator of mitochondrial fission: dynamin-related protein 1 (Drp1), along with fission adaptor proteins, mitochondrial fission factor and Fis1 (Friedman et al., 2011; Moltedo et al., 2019). Fusion of mitochondria require the involvement of MFN1 and MFN2 which, as aforementioned, are enriched at MAMs and regulate MAM tethering (Moltedo et al., 2019). Notably, MFN1/2 coordinated OMM fusion while optic atrophy protein 1 (OPA1) mediated IMM fusion.

Beyond fission and fusion, biogenesis and degradation are also vital for ensuring healthy mitochondria turnover. Following mitochondrial fission events, damaged mitochondria are removed *via* mitophagy, in which phosphatase and tensin homolog-induced putative kinase (PINK1) and beclin 1 (BECN1) at the MAM interface promote autophagosome formation (Gelmetti et al., 2017). Notably, PINK1 phosphorylates MFN2 leading to Parkin recruitment and initiation of mitophagy machinery while proteins such as p62 and microtubule-associated protein 1A/1B-light chain 3 (LC3) coordinate cargo selection and autophagosome/mitophagosome maturation (Moltedo et al., 2019; Yang et al., 2020). Meanwhile, biogenesis is a self-renewal process in which mitochondrial machinery is ‘replenished’ by increased transcription and translation of mitochondrial DNA (mtDNA) as well as increased synthesis, import, and assembly of nuclear DNA-encoded mitochondrial proteins (Popov, 2020). While the role of MAMs in mitochondrial biogenesis is not clear, expression of proliferator-activated receptor γ coactivator 1α (PGC1α), a master regulator of mitochondrial biogenesis, controls ER-mitochondrial contact. Notably, PGC1α knockout perturbs MAM contact while overexpression promotes increased interaction (Ciron et al., 2015). Finally, mitochondrial transport is essential to ensure mitochondria are distributed to meet the energetic demands of a cell. Trafficking of mitochondria is facilitated by mitochondrial Rho GTPases (Miro), which directly interact with MFN1/2 and regulate ER-mitochondrial contact (Misko et al., 2010, 2012; Modi et al., 2019). Moreover, functional MFN2 is required for mitochondrial mobility (Misko et al., 2010, 2012).

## Mitochondria-Associated Endoplasmic Reticulum Membranes in Neuropathology

Given the importance of MAM-associated mechanisms in cellular homeostasis, MAMs are gaining attention as plausible pathological platforms underlying the development and/or progression of disease (Pinton, 2018). Briefly, the role of MAMs in neuropathology, which have been previously reviewed (Raeisossadati and Ferrari, 2020; Wilson and Metzakopian, 2020; Leal and Martins, 2021), have primarily been centered on neurons, highlighting MAMs as pivotal regulators of synaptic transmission and neuronal health (Bernard-Marissal et al., 2018; Gomez-Suaga et al., 2019; Shirokova et al., 2020; Leal and Martins, 2021). Astrocyte MAMs are largely a new exploration. Notably, enrichment of MAMs in astrocyte endfeet may be crucial for regulating the blood-brain interface and brain healing following injury (Bergami and Motori, 2020; Gbel et al., 2020). Moreover, knockdown of MAM-associated proteins, PACS2 or σ1R, induces degeneration of hippocampal neurons and astrocytes, supporting the importance of MAMs for neural cell survival (Hedskog et al., 2013). The presence and function of MAMs in other neural cells, microglia and oligodendrocytes, remain unconfirmed (Bernard-Marissal et al., 2018; Shirokova et al., 2020).

Many of the cellular processes that are implicated in the etiology of neurodegenerative diseases are coordinated at the ER-mitochondrial interface such as dysregulated lipid and Ca^2+^ homeostasis, mitochondrial dysfunction, ER and oxidative stress, impaired autophagy, and inflammation. Neurons are particularly susceptible to mitochondrial dysfunction given the high energetic demand of brain tissue (Grimm and Eckert, 2017; Picca et al., 2020). Moreover, ER stress is induced by the aggregation of misfolded proteins, which is a physical hallmark of most neurodegenerative pathologies. Formation of protein aggregates can be a consequence of increased production of misfolded proteins (ER dysfunction), the impaired removal of dysfunctional proteins (impaired autophagy), or both (Sweeney et al., 2017; Monaco and Fraldi, 2020). Regardless, ER stress and mitochondrial dysfunction are increasingly being proposed as key therapeutic targets for combating neuropathology, with MAMs arising as the essential crossroad for this collaboration. It is worth considering whether these central hubs can be manipulated to reconcile cellular dysfunction and degeneration and to restore CNS homeostasis.

**FIGURE 2:**
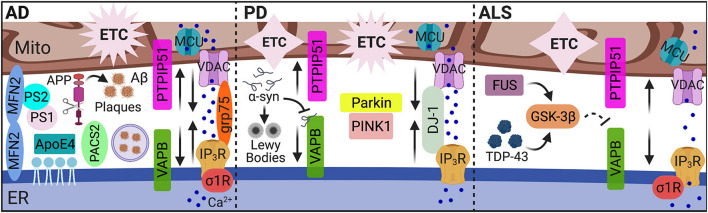
MAMs in AD, PD, and ALS. While dysregulated MAM-associated mechanisms are hallmarks of neurodegenerative pathology, the unique pathological features of AD, PD, and ALS alter MAM contact and communication emphasizing MAMs as pivotal players in neuropathology. In AD, MAM contact, Ca^2+^ transfer, and MAM-mediated mechanisms (i.e., autophagy) are increased among *in vitro* and mouse models, yet decreased MAM-mediator expression and interaction was found in human AD cortical tissues. In PD, both increased and decreased MAM contact and communication were reported. In ALS, MAM tethering and Ca^2+^ transfer were significantly impaired. MAMs, mitochondria-associated ER membranes; Mito, mitochondria; ER, endoplasmic reticulum; AD, Alzheimer’s disease; Aβ, amyloid β; APP, amyloid precursor protein; ApoE4, ε4 allele of apolipoprotein E; PS, presenilin; MFN, mitofusin; PACS2, phosphofurin acidic cluster sorting 2; Ca^2+^, calcium; IP_3_R, inositol 1,4,5-triphosphate receptors; VDAC, voltage-dependent anion-selective channel; grp75, glucose-regulated protein 75 kDa; MCU, mitochondrial Ca^2+^ uniporter; σ1R, sigma-1 receptor; ETC, electron transport chain; PD, Parkinson’s disease; α-syn, α-synuclein; VAPB, vesicle-associated membrane protein-associated protein B; PTPIP51, protein tyrosine phosphatase-interacting protein 51; PINK1, phosphatase and tensin homolog-induced putative kinase; ALS, amyotrophic lateral sclerosis; TDP-43, transactive response (TAR) DNA-binding protein 43; GSK-3β, glycogen synthase kinase-3β; and FUS, fused in sarcoma. Image created with BioRender.com.

Beyond the classic hallmarks of neuropathology in the context of known MAM-associated functions, dysregulation of MAM tethering and activity are implicated in a number of neurodegenerative diseases such as Alzheimer’s disease (AD), Parkinson’s disease (PD), and amyotrophic lateral sclerosis (ALS). However, whether these MAM alterations arise as intended remedial responses or are the causative agents in these disease pathologies remains to be determined. The distinct interplay of MAMs in these neuropathological conditions is illustrated in Figure 2 and discussed below.

### Alzheimer’s Disease

As one of the most common forms of dementia, Alzheimer’s disease is characterized by the aggregation of extracellular amyloid β (Aβ) peptides and intracellular hyperphosphorylated tau proteins, termed plaques and tangles, respectively. Treatment with Aβ in primary hippocampal neurons increased expression of MAM-associated PACS2 and σ1R proteins, as well as direct ER-mitochondrial contact *via* the IP_3_R-VDAC bridging complex. Expression of PACS2 and σ1R was also increased in the hippocampus, cortex, and cerebellum brain regions of a potent amyloid precursor protein (APP) mutant AD mouse model. However, in human AD postmortem cortical tissues, PACS2 increased, but σ1R expression decreased (Hedskog et al., 2013). Interestingly, MAMs serve as a key production site for Aβ peptides (Schreiner et al., 2015). The formation of Aβ plaques occurs following the catalytic processing of APP by the γ-secretase complex. Two major components of this complex, presenilin (PS) 1 and PS2, localize to the ER-mitochondria interface and modulate MAM functions, specifically lipid and Ca^2+^ homeostasis (Area-Gomez et al., 2009; Zampese et al., 2011; Area-Gomez et al., 2012; Galla et al., 2020). Notably, overexpressing PS2 increases both ER-mitochondria contact and Ca^2+^ transfer from the ER to mitochondria (Zampese et al., 2011; Galla et al., 2020). This phenomenon appears to be mediated through interactions between PS2 and MFN2 (Filadi et al., 2016). Furthermore, silencing MFN2 in human embryonic kidney (HEK) 293 cells stably expressing an APP mutant to overproduce Aβ, increased MAM contact and Ca^2+^ transfer, which impairs γ-secretase maturation and activity, ultimately decreasing Aβ production (Leal et al., 2016). Leal et al. also recently reported decreased expression of MFN1/2 in postmortem human AD brain tissues. They further confirmed a positive correlation between Aβ and MAM contact in multiple AD mouse models and *in vitro* neuron cultures exposed to Aβ. The increased connectivity between ER and mitochondria in response to Aβ subsequently increased mitochondrial metabolic function and autophagosome formation, likely to promote removal of Aβ aggregates (Leal et al., 2020). Of note, another recent examination of human AD cortical tissues showed a decreased expression and interaction of MAM tether proteins, VAPB and PTPIP51, in addition to decreased IP_3_R expression. The decreased expression of these proteins also correlated with increased disease stage severity (Lau et al., 2020). Thus, decreased expression of MAM-mediators in human AD tissues was reported across three separate studies (Hedskog et al., 2013; Lau et al., 2020; Leal et al., 2020) suggesting impaired ER-mitochondrial tethering, highlighting unique differences between animal models and human tissues. Regardless, these findings strongly implicate MAMs as possible targets to combat AD/Aβ pathology.

Disease-associated tau protein alters mitochondrial transport, dynamics, bioenergetics and degradation (reviewed by Szabo et al., 2020). These findings further discuss the implications of MAMs in tau pathology as only two studies have so far explored this phenomenon; both of these models identified increased ER-mitochondrial contact during tau pathology (Perreault et al., 2009; Cieri et al., 2018). The ε4 allele of apolipoprotein E (ApoE4) significantly associates with an increased risk for sporadic AD. Treatment of astrocyte conditioned media cultured from ApoE4 knock-in mice, upregulated MAM activity in human fibroblasts and mice neurons, as measured by the synthesis of phospholipids and cholesteryl esters (Tambini et al., 2016). This outcome was averted when repeated in mouse embryonic fibroblasts (MEF) harboring MFN2 knockout, which in this model, decreased MAM tethering (de Brito and Scorrano, 2008; Tambini et al., 2016).

### Parkinson’s Disease

Parkinson’s disease is a neurodegenerative disease manifesting with primarily motor deficits due to damaged and degenerative dopaminergic neurons in the substantia nigra. A key pathophysiology underlying PD is the presence of ‘Lewy bodies’ comprised of α-synuclein (α-syn) aggregates. However, tau pathology is also present in some cases. PD pathology is characterized by a number of MAM-associated cellular processes including impaired autophagy, Ca^2+^ homeostasis, lipid metabolism, ER stress, and mitochondrial dynamics (reviewed by Rodriguez-Arribas et al., 2017; Gomez-Suaga et al., 2018). Notably, overexpression or silencing of α-syn confirms regulation on mitochondrial dynamics, ER-mitochondrial coupling and Ca^2+^ transfer (Cali et al., 2012; Guardia-Laguarta et al., 2014). However, these findings are also inconsistent as one demonstrates increased organelle contact while the other reports disruption in tethering. From a more recent report, α-syn binds to VAPB on the ER membrane, disrupting interaction with PTPIP51 on the OMM and subsequently, MAM tethering, Ca^2+^ transfer, and ATP production (Paillusson et al., 2017). Thus, the effects of α-syn on MAM function are linked to impaired bioenergetic activity.

Another MAM connection to the neuropathology of PD involves PINK1 and Parkin. Mutations in PINK1 and Parkin are key risk factors for the development of PD. The functions of these proteins are critical in maintaining mitochondrial health by regulating mitochondrial biogenesis, degradation, dynamics, function, and transport. PINK1 and Parkin localize to MAMs, which is not surprising given their prominent roles in mitophagy and MAM’s being the site for mitochondrial fission (Gomez-Suaga et al., 2018). Moreover, Parkin is implicated as a possible regulator for MAM tethering although controverting evidence has been reported as to whether it is a positive or negative regulator (Cali et al., 2013; Gautier et al., 2016). Briefly, overexpression of Parkin in both HeLa and a neuroblastoma cell line enhanced MAM coupling and Ca^2+^ transfer to increase ATP production (Cali et al., 2013). Expression of Parkin is encoded by the *PARK2* gene. Increased mitochondrial-ER contact was found in fibroblasts from a *PARK2* knockout mouse and PD patients with *PARK2* mutation (Gautier et al., 2016). Moreover, mutations in the gene for DJ-1, which as mentioned above has been identified as a novel component in the IP_3_R-grp75-VDAC complex, are causative for autosomal recessive familial PD. The DJ-1 protein has been identified to have a protective role in oxidative stress and modulates mitochondrial morphology. Similar to a report on α-syn, overexpression of DJ-1 enhances ER-mitochondrial coupling and Ca^2+^ transfer while silencing of DJ-1 impairs mitochondrial Ca^2+^ flux and induces fragmentation (Ottolini et al., 2013). All these findings highlight the importance of MAMs as regulators of mitochondrial function and physiology in the context of PD pathology.

### Amyotrophic Lateral Sclerosis

A key component of ALS pathology is deposits of transactive response (TAR) DNA-binding protein 43 (TDP-43). Like α-syn, TDP-43 alters MAM tethering and Ca^2+^ homeostasis by interrupting the relationship between VAPB and PTPIP51. However, the mechanism of disruption is instead mediated through activation of glycogen synthase kinase-3β (GSK-3β) (Stoica et al., 2014). Interestingly, accumulation of fused in sarcoma (FUS), a key pathological feature characterizing ALS, also interrupts VAPB and PTPIP51 association and MAM tethering *via* GSK-3β activation (Stoica et al., 2016). This interruption was accompanied by compromised mitochondrial Ca^2+^ uptake and ATP production. Thus, ALS pathology is characterized by two factors that activate GSK-3β to disentangle the MAM interface; however, the target of GSK-3β to facilitate this interference remains unknown. The significance of MAM tethering in ALS pathology is further supported as a P56S mutation in VAPB is causative of ALS (Nishimura et al., 2004). Moreover, mutation or perturbed function of another MAM protein, σ1R, which is essential for Ca^2+^ transfer from the ER to mitochondria, is a causal agent to ALS pathology and motor neuron degeneration (Al-Saif et al., 2011; Bernard-Marissal et al., 2015). Additional implications and associations of MAMs in ALS pathology are previously reviewed (Manfredi and Kawamata, 2016; Lau et al., 2018).

## Human Immunodeficiency Virus- Associated Neurocognitive Disorders

Human immunodeficiency virus type 1 (HIV-1) can invade the CNS early during infection and infect residential glial cells (astroglia, microglia, and oligodendrocytes) where infection can persist for life. Even with the medical advancement of antiretroviral therapy (ART), low viral replication, chronic neuroinflammation, glial dysfunction, and HIV-1 protein toxicity contribute to the development of a spectrum of HIV-associated neurocognitive disorders (HAND). In fact, HAND continue to afflict approximately 30–70% of people living with HIV, depending on cohort demographics (Simioni et al., 2010; Heaton et al., 2011). HAND are characterized by different levels of cognitive impairments and interference with one’s daily functioning. At the extreme end of the spectrum, symptoms clinically manifest as dementia (Mackiewicz et al., 2019).

The pathology of HIV-1 infection often includes “accelerated aging.” Thus, HIV-1 patients are more prone to developing early onset symptoms for a number of age-related diseases including the neurodegenerative pathologies discussed above (Robertson et al., 2007; Cody and Vance, 2016; Wang et al., 2017; Mackiewicz et al., 2019). In fact, Aβ plaques as well as tauopathy can both associate with and be exacerbated by HAND pathology (Kim et al., 2013; Brown et al., 2014; Hategan et al., 2019). Of note, the primary HIV-1 proteins implicated in neurotoxicity are transactivator of transcription (Tat), glycoprotein (gp)120, viral protein R (Vpr), and negative factor (Nef). Moreover, ART drugs are now identified as key contributors to the cellular senescence and accelerated aging underlying HIV/HAND pathology, which was reviewed previously with an emphasis on mitochondrial dysfunction (Schank et al., 2021).

**FIGURE 3:**
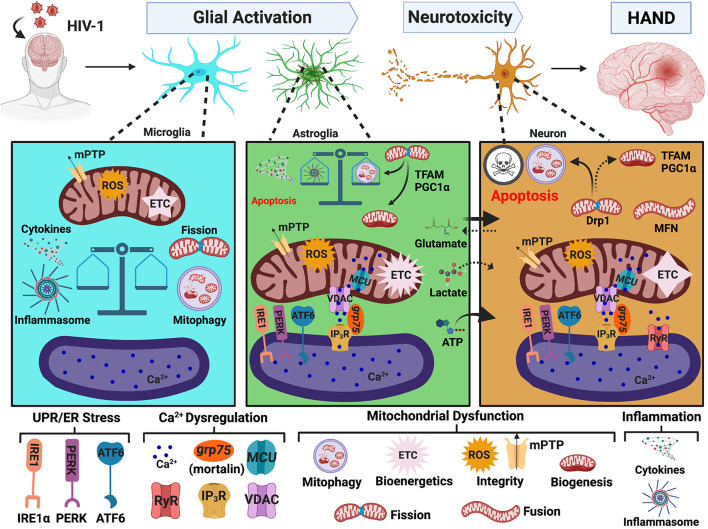
MAMs in HAND. HIV-1 in the CNS leads to glial activation and neurotoxicity, which are key mechanisms mediating HAND pathology. HIV-induced UPR/ER stress, Ca^2+^ dysregulation, and mitochondrial dysfunction strongly implicate MAMs as potential regulators of glial activation and neurotoxicity. In microglia, the initiation of mitophagy is essential to buffer HIV-induced mitochondrial dysfunction but accumulation of damaged mitochondria induces a heightened inflammatory response which is associated with impaired mitochondrial integrity and a significant decrease in bioenergetic capacity. Conversely, astrocytes increase their Ca^2+^ signaling and metabolic capacity. Astrocyte shift in function leads to increased release of neurotoxic factors and well as impaired provision of essential nutrients to neurons. Similar to microglia, a balance between mitophagy and inflammasome activation appears to be a critical determinate to astrocyte fate. Notably, ER-mitochondrial Ca^2+^ transfer and UPR/ER stress also arise as potential pivotal players in astrocyte-mediated neurotoxicity. While astrocytes and microglia teeter between toxic and tropic, neurons are highly vulnerable to HIV-induced mitochondrial dysfunction caused by both direct and indirect insults. Targeting MAMs to combat mitochondrial dysfunction is of heightened importance to enhance CNS fitness against neuropathologic challenges, including HIV/HAND. MAMs, mitochondria-associated ER membranes; HIV-1, human immunodeficiency virus type 1; HAND, HIV-associated neurocognitive disorders; ER, endoplasmic reticulum; UPR, unfolded protein response; PERK, protein kinase RNA-like endoplasmic reticulum kinase; IRE1, inositol-requiring protein 1α; ATF6, activating transcription factor 6; Ca^2+^, calcium; IP_3_R, inositol 1,4,5-triphosphate receptors; VDAC, voltage-dependent anion-selective channel; grp75, glucose-regulated protein 75 kDa; MCU, mitochondrial Ca^2+^ uniporter; RyR, ryanodine receptors; MFN, mitofusin; Drp1, dynamin-related protein 1; mPTP, mitochondrial permeability transitional pore; ROS, reactive oxygen species; ETC, electron transport chain; PGC1α, proliferator-activated receptor γ coactivator 1α; TFAM, transcription factor A. Image created with BioRender.com.

Khan et al. (2019) recently published insights into potential inter-organelle collaborations in HIV/HAND pathogenesis. Despite obvious disruptions in ER and mitochondrial homeostasis in HAND pathology, there remains limited investigations and/or considerations of MAMs (Ma et al., 2016; Nooka and Ghorpade, 2017; Khan et al., 2019). Below we discuss the implications of MAMs in HAND pathology by reviewing the effects of HIV-1 on ER stress, Ca^2+^ dysregulation and mitochondrial dysfunction, which is also illustrated in Figure 3. Together, these findings strongly support altered MAM signaling as a prominent contributor to HAND pathology.

### Human Immunodeficiency Virus-1 and Endoplasmic Reticulum Stress

As aforementioned, ER stress is a key characteristic in neurodegenerative pathologies, and MAMs are intricately involved in cellular responses to ER stress. Indeed, the three UPR arms are now increasingly considered integral mediators within the MAM proteome (van Vliet and Agostinis, 2016, 2018; Saito and Imaizumi, 2018; Malli and Graier, 2019). In the context of HAND, significant increases in ER stress markers are detected in the frontal cortex of brain tissues from HIV-1 positive individuals. In fact, the levels of ER stress positively correlate with cognitive decline, wherein the most severe cases of HAND have the highest expression of ER stress markers (Lindl et al., 2007; Akay et al., 2012). Interestingly, these studies show that while ER stress is evident in both neurons and astrocytes, astrocytes appear to have higher expression of ER stress markers. These findings do not necessarily mean neurons are less likely to endure ER stress during HIV-1 infection. Rather, astrocytes may be more resilient to prolonged ER stress, and neurons could be more susceptible to succumb to apoptosis.

It is well-known that during neurodegenerative or neuroinflammatory conditions, astrocytes become ‘activated’, shifting their function from neurotrophic to a more neurotoxic phenotype. Astrocyte-mediated neurotoxicity has arisen as a key feature in HAND pathology with UPR/ER stress as a potential regulator (Fan and He, 2016; Shah et al., 2016; Nooka and Ghorpade, 2017, 2018; Natarajaseenivasan et al., 2018). In fact, while HIV-1-induced ER stress has not yet been extensively investigated, most investigations are primarily centered around astrocytes. Notably, expression of HIV-1 Tat in astrocytes induces aggregation of glial fibrillary acid protein (GFAP). Both Tat expression and GFAP aggregation activate the three UPR pathways (Fan and He, 2016). HIV-1 gp120 specifically activates the IRE1α branch of UPR signaling in an astrocyte cell line, primary human astrocytes, and astrocyte-restricted gp120 transgenic mice, which is linked to the initiation of apoptotic signaling (Shah et al., 2016). Inhibition of ER stress or UPR signaling in both of these studies reverses astrocyte-mediated neurotoxicity and apoptotic signaling (Fan and He, 2016; Shah et al., 2016). Moreover, studies from our lab demonstrate induction of the three UPR pathways in primary human astrocytes in response to HAND-relevant stimuli (whole HIV-1, inflammation, and ART drugs), which associate with mitochondrial depolarization (Nooka and Ghorpade, 2017). HIV-1 Tat-induced ER stress in a CD4+ T cell line (Campestrini et al., 2018) neurons (Norman et al., 2008) and human brain microvascular endothelial cells (HBMEC) (Ma et al., 2016) are also accompanied by changes in mitochondrial function and apoptotic signaling supporting cooperation of the ER and mitochondria during HIV/HAND pathology. Indeed, inhibition of ER stress reversed HIV-1-induced mitochondrial dysfunction and increased cell viability in HBMEC (Ma et al., 2016). These findings not only implicate MAMs in HAND pathology but also emphasize UPR/ER stress signaling as an important regulator of mitochondrial function, cellular fate and astrocyte-mediated neurotoxicity.

### Human Immunodeficiency Virus-1 and Ca^2+^ Dysregulation

As discussed above, the ER is the primary site for intracellular Ca^2+^ storage, and MAMs are hubs for regulating Ca^2+^ homeostasis. HIV-1 modulates Ca^2+^ signaling and perturbs Ca^2+^ homeostasis. Many of these studies have been previously reviewed or discussed (Haughey and Mattson, 2002; Hu, 2016; Fields and Ellis, 2019). Altered Ca^2+^ dynamics during HIV-1 exposure is directly linked to apoptotic signaling, UPR induction, and impaired mitochondrial integrity, energetics, and/or quality control thus implicating Ca^2+^ dysregulation as a key component in HIV-1-induced neurotoxicity and astrogliosis. One of the earliest studies identifying this relationship was in rat hippocampal neurons exposed to HIV-1 Tat, which induced apoptosis through the elevation of cytoplasmic Ca^2+^ and increased mitochondrial Ca^2+^ uptake. Chelating cytosolic Ca^2+^ or pharmacological inhibiting mitochondrial Ca^2+^ uptake *via* MCU protected neurons from HIV-1 Tat-mediated neurotoxicity (Kruman et al., 1998). Interestingly, a later study in rat cortical neurons linked ER and mitochondrial Ca^2+^ loss *via* ryanodine receptors (RyR) as an upstream mechanism for HIV-1 Tat-mediated UPR induction and mitochondrial hyperpolarization (Norman et al., 2008). While less prominent, Ca^2+^ mobilization is also implicated in HIV-1 gp120-(Meeker et al., 2016) and Vpr-(Jones et al., 2007) mediated neurotoxicity, which associated with impaired axonal mitochondria transport and apoptotic signaling, respectively.

In the context of astrocytes, our previous studies indicate a pivotal role of Ca^2+^ signaling in regulating ER stress and mitochondrial dysfunction induced by HAND-relevant stimuli (whole HIV-1, inflammation, and ART drugs). Notably, chelation of cytoplasmic Ca^2+^ was able to reverse HIV-1-induced ER stress and mitochondrial depolarization (Nooka and Ghorpade, 2017). Moreover, knockdown of MCU to reduce mitochondrial Ca^2+^ uptake during exposure to HIV-1 Tat and/or cocaine reverses astrocyte mitochondrial dysfunction and metabolic switching to restore a neuroprotective phenotype (Natarajaseenivasan et al., 2018). Manipulation of VDAC1 (the OMM Ca^2+^ channel within the MAM interface) or “mortalin” (aka grp75, the scaffolding protein between IP_3_R on the ER membrane and VDAC on the mitochondria) is also able to rescue neurons from HIV-1 Tat-induced astrocyte-mediated neurotoxicity (Fatima et al., 2017; Priyanka et al., 2020). Specifically, HIV-1 Tat expressing primary human astrocytes trigger neuronal death by excessive ATP release, a mechanism that was counteracted by repression of VDAC1 (Fatima et al., 2017). Neuroinflammation and glutamate excitotoxicity are additional mechanisms for which astrocytes inflict neuronal damage during HAND, as previously reviewed by our research team (Cisneros and Ghorpade, 2012; Borgmann and Ghorpade, 2015). Overexpression of mortalin/grp75 protects neurons from astrocyte-mediated neurotoxicity by reversing HIV-1 Tat-induced astrocyte mitochondrial dysfunction and fragmentation while also reducing the release of excess ATP, inflammatory cytokines, and extracellular glutamate (Priyanka et al., 2020). Altogether, these findings strongly support MAM-mediated Ca^2+^ transfer as a pivotal regulator of astrocyte-mediated neurotoxicity during HAND pathogenesis.

It is also noteworthy to mention, that while research with HIV-1 Nef is less investigated in HAND pathogenesis, it is known to play a prominent role for Ca^2+^ dysregulation in T cells (Manninen and Saksela, 2002; Shelton et al., 2012; Silva et al., 2016). In fact, HIV-1 Nef directly interacts with both mortalin/grp75 (Shelton et al., 2012) and IP_3_R (Manninen and Saksela, 2002), which are key mediators in ER to mitochondria Ca^2+^ transfer. Thus, there is likely an interplay between HIV-1 Nef and the MAM interface. More studies are needed to determine the role of HIV-1 Nef and Ca^2+^ dysregulation in neural cells.

### Human Immunodeficiency Virus-1 and Mitochondrial Dysfunction

The MAMs are essential for regulating mitochondrial function and homeostasis. Defects in mitochondrial bioenergetics, biogenesis, degradation, dynamics, integrity and transport are all present in the pathology of HAND and are discussed below. The role of mitochondrial dysfunction in HIV/HAND pathology is rapidly gaining attention with two recent reviews by Fields and Ellis (2019) and Schank et al. (2021); however, neither discuss the potential interplay of MAMs. It is well established that HIV-1 can induce neuronal apoptosis, in which mitochondria [and MAMs] are known upstream regulators through Ca^2+^ and redox signaling (Kruman et al., 1998; van Vliet et al., 2014; Marchi et al., 2017). In addition to regulating apoptosis, Ca^2+^ and redox signaling between mitochondria and ER are also essential for regulating mitochondrial integrity and bioenergetic activity, autophagy and inflammasome activation (van Vliet et al., 2014), again highlighting the importance of ER-mitochondrial inter-organelle collaboration in negotiating cellular fate.

In human neurons, exposure to HIV-1 Tat and Vpr decreased VDAC (OMM Ca^2+^ transporter) protein expression, dysregulated RNA expression of several genes regulating mitochondrial metabolism and decreased ATP levels (Darbinian et al., 2020). These findings were also associated with mtDNA damage, reactive oxygen species (ROS) accumulation, increased expression apoptotic proteins, and release of cytochrome C, emphasizing the relationship of mitochondrial bioenergetics and integrity. Less clear are the effects of HIV-1 on neuronal mitochondrial dynamics, which vary greatly across investigations. Two separate studies looking at the brain tissues from HIV + donors with or without HIV encephalitis, specifically centered on neurons, report enlarged mitochondria as a unique pathological feature of HAND (Avdoshina et al., 2016; Fields et al., 2016).

Changes in mitochondrial morphology associated with increased expression of fusion proteins (MFN1 and OPA1) and decreased expression and/or activation of mitochondrial fission proteins (Fis1 and Drp1). Interestingly, as previously discussed with ER stress markers, decreased expression of mitochondrial Drp1 also coincided with increased HIV-associated neurocognitive decline (Fields et al., 2016). Similar disruptions on mitochondrial morphology were confirmed in gp120 transgenic mice and by gp120 treatment to neuronal cell cultures (Avdoshina et al., 2016; Fields et al., 2016). These reports also found increased extracellular acidification rate indicating increased glycolysis (Fields et al., 2016) and reduced oxygen consumption rate (OCR) (Avdoshina et al., 2016) suggesting a deviation in mitochondrial bioenergetics. Noteworthy, overexpressing Drp1 reverses gp120-meditated neuronal mitochondrial dysfunction reducing both neuroinflammation and neurodegeneration (Fields et al., 2016). These findings suggest mitochondrial fission as a potential therapeutic mechanism to combat HIV-mediated mitochondrial dysfunction in neurons.

On the contrary, more recent studies by Teodorof-Diedrich and Spector (2018) looking at the effects of both HIV-1 gp120 and Tat on mitochondrial dynamics in human neurons and Rozzi et al. (2018) looking at the effects of HIV-1 Tat in rat cortical neurons, instead identified a proclivity toward mitochondrial fission and fragmentation. In fact, increased Drp1 expression, activity, and/or translocation were critical for these outcomes. Both studies also reported impaired mitochondrial integrity as demonstrated by decreased membrane potential (ΔΨm). Noteworthy, Teodorof-Diedrich and Spector (2018) also found an increased recruitment of mitophagy proteins (Parkin, p62, and LC3) and mitophagosome formation, yet an impaired mitophagic flux leading to accumulation of damaged mitochondria due to incomplete mitophagy. The discrepancy regarding HIV-1 gp120 on neuronal mitochondrial dynamics may be attributed to different *in vitro* models as the prior two studies were in rodents (Avdoshina et al., 2016; Fields et al., 2016) and the later in primary human neurons (Teodorof-Diedrich and Spector, 2018). Moreover, there may be general discrepancies between the effects of HIV-1 gp120 versus HIV-1 Tat. Regardless, the identical findings of elongated mitochondria in neurons of HIV+ brain tissues remain a significant find.

However, there is one similarity across these four studies in regard to mitochondrial distribution/localization. There appears to be an overall decrease in mitochondrial trafficking throughout the processes with increased aggregation of mitochondria near the soma. Impaired mitochondria axonal transport is also evident in neurons exposed to HIV-1 Vpr, which was further associated with decreased ATP production and increased expression of senescent markers (Wang et al., 2017). As mitochondria are essential for synaptic maintenance and to meet the energetic demands for neurotransmission, disrupted axonal mitochondria transport arises as a potential key pathological feature in HAND and age-related axonal degeneration. Interestingly, although HIV-1 Vpr is primarily known as a nuclear protein, it also directly interacts with adenine nucleotide translocase (ANT) on the OMM, which is a key regulator of mitochondrial integrity *via* formation of the mitochondrial permeability transitional pore (Huang et al., 2012; Wang et al., 2017; Cowan et al., 2019). In fact, inhibiting the interaction between HIV-1 Vpr and ANT was able to reverse deficits in mitochondrial trafficking (Wang et al., 2017). Finally, this study also suggests HIV-1 Vpr may disrupt mitochondrial biogenesis in neurons *via* reduction of PGC1α expression. Swinton et al. (2019) confirmed decreased expression of PGC1α in HIV+ brain cortical tissues as well as decreased transcription factor A (TFAM), another regulator of mitochondrial biogenesis (Swinton et al., 2019).

In astrocytes, both HIV-1 infection and external HIV-1 exposure perturb mitochondrial integrity (Nooka and Ghorpade, 2017; Natarajaseenivasan et al., 2018; Ojeda et al., 2018; Priyanka et al., 2020). Unlike neurons, astrocytes increase their metabolic activity and ATP production (Natarajaseenivasan et al., 2018; Swinton et al., 2019). Moreover, while neurons have decreased TFAM expression in HIV+ brain tissues, astrocytes have increased TFAM expression (Swinton et al., 2019). The increased metabolic and mitochondrial biosynthetic profile may be a key feature underlying astrocyte activation and astrocyte-mediated neurotoxicity during HAND. For example, HIV-1 Tat provokes astrocytes to undergo a distinct metabolic shift from glucose to fatty acid oxidation, which restricts astrocyte provision of lactate to neurons (Natarajaseenivasan et al., 2018). In addition to decreasing release of neurotrophic factors when ‘activated’ by HIV-1 Tat, astrocytes increase their release of neurotoxic factors including excessive ATP (Fatima et al., 2017; Priyanka et al., 2020), ROS (Natarajaseenivasan et al., 2018; Priyanka et al., 2020), and inflammatory cytokines (Natarajaseenivasan et al., 2018). As briefly discussed in the previous section, blocking ER-mitochondrial Ca^2+^ transfer *via* VDAC (on the OMM) (Fatima et al., 2017), MCU (on the IMM) (Natarajaseenivasan et al., 2018), or mortalin/grp75 (scaffold between IP_3_R and VDAC) (Priyanka et al., 2020) restored an astrocyte neurotrophic phenotype, highlighting a probable MAM interplay in astrocyte-mediated neurotoxicity during HAND. Moreover, HIV-1 infection (Ojeda et al., 2018) or HIV-1 Tat expression (Priyanka et al., 2020) in astrocytes induced mitochondrial fragmentation, which may be followed by defective mitophagy, similar to what was seen by Teodorof-Diedrich and Spector (2018) in neurons. Notably, accumulation of damaged mitochondria promoted inflammasome activation, which was subsequently followed by cell death (Ojeda et al., 2018). However, astrocytes that had successful mitophagy, were able to attenuate mitochondrial dysfunction and resist cell death. The divergence between these fates appeared dependent on the mode of infection, where productively infected astrocytes favored survival, and non-productive infection succumbed to inflammasome-mediated cell death.

Mitochondrial dysfunction in microglia has been less studied; however, there is a recent review discussing the important role of microglia in HAND (Borrajo et al., 2021). Similar to astrocytes, there appears to be a delicate balance between mitophagy and inflammasome formation underlying microglia activation during HAND (Thangaraj et al., 2018; Rawat et al., 2019). In response to HIV-1 Tat (Thangaraj et al., 2018) or ssRNA (Rawat et al., 2019), there is increased expression of autophagy/mitophagy proteins (PINK1, Parkin, p62, LC3, and BECN1) with a subsequent blockade of mitophagy flux, leading to the accumulation of mitophagosomes and damaged mitochondria. These changes are associated with increased ROS generation and impaired mitochondrial integrity (Rawat et al., 2019). However, unlike astrocytes, there is a significant decrease in mitochondrial bioenergetic activity (Thangaraj et al., 2018). Importantly, defects in mitophagy appear to be central to microglial activation and inflammasome formation (Thangaraj et al., 2018; Rawat et al., 2019). Indeed, HIV-1 gp120 induces inflammasome activation in microglia *in vitro* and *in vivo*, whereas inhibiting inflammasome activation, alleviates microglia-mediated neurotoxicity, promotes neuronal regeneration, and improves cognitive function (He et al., 2020).

In summary, HIV-induced changes in mitochondrial bioenergetics, dynamics, degradation, integrity, and transport are MAM-regulated processes, implicating MAMs in HIV/HAND pathology. Noteworthy, mitochondrial fission events are key consequences of Tat-mediated toxicity in neurons (Rozzi et al., 2018; Teodorof-Diedrich and Spector, 2018), astrocytes (Ojeda et al., 2018; Priyanka et al., 2020), and microglia (Thangaraj et al., 2018; Rawat et al., 2019). Further, defective mitophagy is a crucial mechanism underlying HAND pathology. The accumulation of damaged mitochondria promotes inflammasome activation in astrocytes and microglia. Thus, the balance between mitophagy and inflammasome activation is a critical determinant of glial fate during HIV-1 toxicity (Ojeda et al., 2018; Rawat et al., 2019). As MAMs are the site for both mitophagy and inflammasome initiation, ER-mitochondrial cooperation is likely essential for negotiating these cellular outcomes. Finally, evidence of MAMs in HAND is further supported by a previous study in T cells showing that HIV-1 Vpr localizes to both the ER and mitochondria, and MAMs serve as a possible route for intracellular trafficking of Vpr (Huang et al., 2012). Notably, exposure to HIV-1 Vpr decreased MFN2 and Drp1 expression, impaired ER-mitochondrial interaction and morphology, and induced mitochondrial depolarization and deformation. Overexpressing MFN2 or Drp1 was able to prevent T cell mitochondrial depolarization and deformation. Additional studies in neural cells are needed to determine the role of MAMs in HAND. Moreover, HIV-1 Tat and Vpr are historically considered as nuclear proteins; however, Tat and Vpr toxicity on ER and mitochondrial homeostasis emphasizes the need to expand our classical understanding of host-viral interactions during HIV-1 infection.

## Potential Therapeutic Targets

Dysfunctional MAM-mediated mechanisms are hallmarks of neurodegenerative pathologies including AD, PD, ALS, and HAND. Notably, ER-mitochondrial contact and communication are critical in regulating mitochondrial function and health. Throughout this review, we have specifically highlighted Ca^2+^, UPR/ER stress, mitochondrial fission/fusion, mitophagy and inflammasome signaling pathways as potential targets to combat mitochondrial dysfunction in neural cells during HIV/HAND, whereas MAMs serve as the central therapeutic platform. Table 1 summarizes the potential therapeutic targets discussed throughout this manuscript.

**TABLE 1:** Potential therapeutic targets.

**Manuscript**	**Model**	**Cell type**	**Target**	**Outcome**
Garrido-Maraver et al., 2020	Drosophila	Drosophila	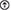 ER-mitochondrial contact	Extended Drosophila lifespan and improved overall motor function
Leal et al., 2016	APP mutant	HEK 293 cells	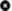 MFN2	Increased MAM contact and Ca^2^**^+^** transfer and decreased Aβ production
Huang et al., 2012	HIV Vpr expression	T cells	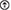 MFN2 and Drp1	Restored mitochondria integrity and morphology and increased cell viability
Kruman et al., 1998	HIV Tat exposure	Neurons	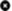 Cytosolic Ca^2^**^+^** and MCU	Protected neurons for HIV-1 Tat-mediated neurotoxicity
Norman et al., 2008	HIV Tat exposure	Neurons	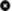 RyR	Attenuated UPR induction and mitochondrial dysfunction
Fields et al., 2016	HIV+ brain tissues, Tg-gp120 mice, gp120 exposure	Neurons	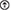 Drp1	Restored mitochondrial dynamics and reduced neuroinflammation (astrogliosis) and neurodegeneration
Rozzi et al., 2018	HIV Tat exposure	Neurons	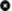 Drp1 (Indirect inhibition)	Prevented Tat-mediated effects on mitochondrial dynamics
He et al., 2020	HIV gp120 exposure; Tg-gp120 mice	Microglia	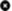 Inflammasome	Reduced neuroinflammation and neurodegeneration. Promoted neuronal regeneration and restored neurocognitive function
Nooka and Ghorpade, 2017	HIV, ART, and IL-1β exposure	Astrocytes	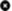 Cytosolic Ca^2^**^+^**	Restored mitochondria integrity
Natarajaseenivasan et al., 2018	HIV Tat exposure	Astrocytes	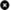 MCU	Reversed metabolic switch and astrocyte-mediated neurotoxicity
Fatima et al., 2017	HIV Tat expression	Astrocytes	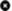 VDAC1	Reversed astrocyte-mediated neurotoxicity
Priyanka et al., 2020	HIV Tat expression	Astrocytes	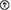 Mortalin/grp75	Reversed astrocyte mitochondrial dysfunction and astrocyte-mediated neurotoxicity
Fan and He, 2016	HIV Tat exposure	Astrocytes	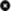 UPR/ER stress	Reversed astrocyte-mediated neurotoxicity and apoptotic signaling
Shah et al., 2016	HIV gp120 exposure; Tg-gp120 mice	Astrocytes	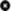 UPR/ER stress	Reversed astrocyte-mediated neurotoxicity and apoptotic signaling
Ma et al., 2016	HIV Tat exposure	HBMECs	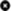 UPR/ER stress	Restored mitochondria integrity and cell viability

*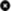 inhibition/knockdown; 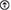 overexpression/upregulation. Aβ, amyloid β; APP, amyloid precursor protein; ART, antiretroviral therapy; Ca^2+^, calcium; Drp1, dynamin-related protein 1; ER, endoplasmic reticulum; gp120, glycoprotein; grp75, glucose-regulated protein 75 kDa; HBMEC, human brain microvascular endothelial cells; HEK, human embryonic kidney; HIV-1, human immunodeficiency virus type 1; IL-1β, interleukin-1β; MFN, mitofusin; MCU, mitochondrial Ca^2+^ uniporter; RyR, ryanodine receptors; Tat, transactivator of transcription; Tg, transgenic; UPR, unfolded protein response; VDAC, voltage-dependent anion-selective channel; Vpr, viral protein R.*

Coupling between the ER and mitochondria can increase in response to stress to tailor to the functional demands of the cell (Bravo-Sagua et al., 2016). However, whether increased/decreased contact contributes to cellular dysfunction or improves cell outcomes, remains largely unknown. Notably, forcing increased ER-mitochondrial contact in Drosophila was able to extend lifespan and improve overall motor function (Garrido-Maraver et al., 2020). As a MAM tether protein, MFN2 may serve as a potential target to manipulate ER-mitochondria contact and communication. Overexpression of MFN2 in CD4+ T cells restored mitochondrial integrity and increased cell viability against HIV-1 Vpr toxicity (Huang et al., 2012). However, the effects of MFN2 manipulation vary across cell types. For example, knockdown of MFN2 decreases MAM contact and communication in MEF (de Brito and Scorrano, 2008) but increases MAM contact and communication in an APP mutant in HEK 293 cells (Leal et al., 2016). It should be noted that increased MAM tethering following MFN2 knockdown decreased Aβ production and improved cellular outcome.

Manipulating MAM tethering can subsequently affect MAM-mediated mechanisms, such as ER to mitochondrial Ca^2+^ transfer. However, with these studies, it becomes difficult to delineate the primary therapeutic mechanism, tethering versus Ca^2+^ transfer. Conversely, studies that directly target MAM-mediated Ca^2+^ transfer provide a clear demonstration of the therapeutic potential of targeting these pathways. For example, in astrocytes exposed to HIV-1, blocking mitochondrial Ca^2+^ uptake by targeting MCU (Natarajaseenivasan et al., 2018), VDAC (Fatima et al., 2017), or cytosolic Ca^2+^ (Nooka and Ghorpade, 2017) can prevent astrocyte mitochondrial dysfunction and reverse astrocyte-mediated neurotoxicity. The same outcome was achieved by overexpression of mortalin/grp75 (Priyanka et al., 2020). However, this was due to a direct interaction of mortalin/grp75 with HIV-1 Tat leading to Tat degradation rather than modulating ER-mitochondrial Ca^2+^ transfer. This is an important discrepancy as mortalin/grp75 manipulation in neurons can protect neurons from oxidative cell death when repressed or increase susceptibility when overexpressed (Honrath et al., 2017). Similarly, blocking MCU or cytosolic Ca^2+^ in neurons improved neuronal survival against HIV-1 Tat toxicity (Kruman et al., 1998), while blocking ER Ca^2+^ release *via* RyR can also attenuate Tat-induced UPR induction and mitochondrial dysfunction (Norman et al., 2008). The potential therapeutic applications of targeting MCU and σ1R to regulate ER-mitochondrial Ca^2+^ transfer to combat neurodegenerative pathologies have also been previously reviewed (Liao et al., 2017; Weng et al., 2017).

Other potential upstream regulators of mitochondrial dysfunction underlying neuropathology are the three UPR/ER stress pathways. In fact, targeting UPR pathways as potential neurodegenerative therapies are previously reviewed (Halliday and Mallucci, 2014; Remondelli and Renna, 2017; Martinez et al., 2019); however, these reports do not focus on UPR/ER stress in the context of mitochondrial dysfunction, regardless of the obvious crosstalk between these two organelles. In the context of HIV/HAND, blocking UPR/ER stress in astrocytes reverses astrocyte-mediated neurotoxicity and apoptotic signaling (Fan and He, 2016; Shah et al., 2016). Similarly, inhibiting UPR/ER stress in HBMECs restores mitochondrial integrity and increases cell viability during HIV-1 Tat toxicity (Ma et al., 2016). It is important to note that there are unique signaling pathways amongst the three arms in addition to their ‘non-canonical’ functions that remain ill-defined. However, given the potential regulation of the UPR/ER stress arms on MAM tethering [PERK/MFN2 (Munoz et al., 2013) and ATF6/VAPB (Gkogkas et al., 2008)], mitochondrial dynamics [PERK (Lebeau et al., 2018) and ATF6 (Bommiasamy et al., 2009)], ER-mitochondrial Ca^2+^ transfer [IRE1α (Carreras-Sureda et al., 2019) and ATF6 (Burkewitz et al., 2020)], and mitochondrial bioenergetics [IRE1α (Carreras-Sureda et al., 2019) and PERK (Balsa et al., 2019)], more research is needed to delineate their therapeutic applications for regulating MAM/mitochondrial dysfunction in neurodegenerative pathologies. Moreover, IRE1α may also regulate mitochondrial dysfunction through inflammasome activation to promote inflammation (Bronner et al., 2015). Indeed, blocking inflammasome signaling in astrocyte-restricted HIV-1 gp120 transgenic mice not only reduced neuroinflammation and neurodegeneration but also promoted neuronal regeneration and restored neurocognitive function (He et al., 2020).

The therapeutic potential of targeting inflammasome signaling gains support when considering the delicate balance between inflammasome activation and mitophagy that is evident in astrocytes and microglia during HIV/HAND. Proceeding mitophagy, mitochondrial fission *via* Drp1 is required for removal of damaged mitochondria, which is essential for cellular survival. Overexpression of Drp1 in astrocyte-restricted HIV-1 gp120 transgenic mice was able to reverse HIV-1 gp120-induced mitochondrial elongation in neurons and reduce neuroinflammation (astrogliosis) and neurodegeneration (Fields et al., 2016). Similarly, Drp1 overexpression in CD4+ T cells expressing HIV-1 Vpr protected mitochondrial integrity and increased cell survival (Huang et al., 2012). Interestingly, cells exposed to HIV-1 Tat increase Drp1 expression and activity favoring mitochondrial fragmentation. Rozzi et al. (2018) was able to prevent fragmentation by indirectly inhibiting Drp1 activation. Thus, while altered mitochondrial dynamics is a notable characteristic of HIV/HAND pathology, the therapeutic application may largely depend on the model/stimuli.

## Concluding Remarks

Cooperation and communication between the ER and mitochondria are essential to ensure maintenance of cellular and organelle homeostasis. The significance of MAMs in different pathologies is indisputable; however, whether MAM dysfunction is a cause or consequence in these pathologies is not yet known. This review presents MAMs as pivotal platforms in neuropathology highlighting specific interplay of MAMs in AD, PD, and ALS. We then discuss the implications of MAMs in HAND for the first time. These reports emphasize Ca^2+^, UPR and inflammasome signaling as potential targets to regulate MAM/mitochondrial dysfunction during neuropathological challenges.

Importantly, the presence and function of MAMs can differ across cell types and pathological conditions. A better understanding of the mechanisms regulating changes in MAMs in a respective cell type and/or pathology is critical to illuminate possible targets for therapeutic manipulation. For example, neurons are not directly infected by HIV-1; thus, indirect HIV-induced toxicity *via* infected glia is a key mechanism in HAND pathology. While glial cells (astrocytes, microglia, and oligodendrocytes) are essential for neural and CNS homeostasis, studies focusing on the presence and function of MAMs in glia are either severely deficient (astroglia) or non-existent (microglia and oligodendrocytes) highlighting the need to investigate ER-mitochondria contact and communication beyond the scope of neurons. Particularly, astrocytes provide essential metabolic and antioxidant support to neurons. Aberrant astrocyte mitochondrial function is a prominent threat to neuronal health and function. Targeting MAMs to manipulate astrocyte mitochondrial function could be a promising avenue to optimize the metabolic and antioxidant coupling between astrocytes and neurons and promote neuronal fitness against CNS pathologies. Moreover, microglia and astrocytes are the residential CNS immune cells participating in neuroinflammation, and MAMs may serve as key hinges for their activated/inflammatory status.

## Author Contributions

JP drafted the manuscript text and figures with guidance and supervision from I-WP and KB. All authors participated in editing or revising the article and agreed to be accountable for the content of the work.

## Conflict of Interest

The authors declare that the research was conducted in the absence of any commercial or financial relationships that could be construed as a potential conflict of interest.

## Publisher’s Note

All claims expressed in this article are solely those of the authors and do not necessarily represent those of their affiliated organizations, or those of the publisher, the editors and the reviewers. Any product that may be evaluated in this article, or claim that may be made by its manufacturer, is not guaranteed or endorsed by the publisher.

## Abbreviations

α-syn: α-synuclein
Aβ: amyloid β
AD: Alzheimer’s disease
ALS: amyotrophic lateral sclerosis
ApoE4: ε4 allele of apolipoprotein E
ANT: adenine nucleotide translocase
APP: amyloid precursor protein
ART: antiretroviral therapy
ATF6: activating transcription factor 6
Bap31: B cell receptor-associated protein 31
BECN1: beclin 1
Ca^2+^: calcium
ER: endoplasmic reticulum
ETC: electron transport chain
Drp1: dynamin-related protein 1
Fis1: fission 1
FUS: fused in sarcoma
GFAP: glial fibrillary acid protein
gp120: glycoprotein 120
grp75: glucose-regulated protein 75 kDa
GSK-3β: glycogen synthase kinase-3β
HBMEC: human brain microvascular endothelial cells
HEK: human embryonic kidney
HIV-1: human immunodeficiency virus type 1
HAND: HIV-associated neurocognitive disorders
IMM: inner mitochondrial membrane
IP_3_R: inositol 1,4,5-triphosphate receptors
IRE1α: inositol-requiring protein 1α
LC3: microtubule-associated protein 1A/1B-light chain 3
MAM: mitochondria-associated ER membrane
MCU: mitochondrial Ca^2+^ uniporter
MEF: mouse embryonic fibroblasts
MFN: mitofusin
Miro: mitochondrial Rho GTPases
mPTP: mitochondrial permeability transitional pore
mtDNA: mitochondrial DNA
OCR: oxygen consumption rate
OMM: outer mitochondrial membrane
Nef: negative factor
OPA1: optic atrophy protein 1
PACS2: phosphofurin acidic cluster sorting 2
PD: Parkinson’s disease
PERK: protein kinase RNA-like endoplasmic reticulum kinase
PGC1α: proliferator-activated receptor γ coactivator 1α
PINK1: phosphatase and tensin homolog-induced putative kinase
PS: presenilin
PTPIP51: protein tyrosine phosphatase-interacting protein 51
ROS: reactive oxygen species
RyR: ryanodine receptors
σ1R: sigma-1 receptor
Tat: transactivator of transcription
TDP-43: transactive response (TAR) DNA-binding protein 43
TFAM: transcription factor A
UPR: unfolded protein response
VAPB: vesicle-associated membrane protein-associated protein B
VDAC: voltage-dependent anion-selective channel
Vpr: viral protein R.

## References

[B1] Akay C. Lindl K. A. Shyam N. Nabet B. Goenaga-Vazquez Y. Ruzbarsky J. (2012). Activation status of integrated stress response pathways in neurones and astrocytes of HIV-associated neurocognitive disorders (HAND) cortex. *Neuropathol. Appl. Neurobiol.* 38 175–200. 10.1111/j.1365-2990.2011.01215.x 21883374 PMC3708539

[B2] Al-Saif A. Al-Mohanna F. Bohlega S. (2011). A mutation in sigma-1 receptor causes juvenile amyotrophic lateral sclerosis. *Ann. Neurol.* 70 913–919. 10.1002/ana.22534 21842496

[B3] Area-Gomez E. De Groof A. J. Boldogh I. Bird T. D. Gibson G. E. Koehler C. M. (2009). Presenilins are enriched in endoplasmic reticulum membranes associated with mitochondria. *Am. J. Pathol.* 175 1810–1816. 10.2353/ajpath.2009.090219 19834068 PMC2774047

[B4] Area-Gomez E. Del Carmen Lara Castillo M. Tambini M. D. Guardia-Laguarta C. De Groof A. J. Madra M. (2012). Upregulated function of mitochondria-associated ER membranes in Alzheimer disease. *EMBO J.* 31 4106–4123. 10.1038/emboj.2012.202 22892566 PMC3492725

[B5] Avdoshina V. Fields J. A. Castellano P. Dedoni S. Palchik G. Trejo M. (2016). The HIV protein gp120 alters mitochondrial dynamics in neurons. *Neurotox. Res.* 29 583–593. 10.1007/s12640-016-9608-6 26936603 PMC4821687

[B6] Balsa E. Soustek M. S. Thomas A. Cogliati S. Garcia-Poyatos C. Martin-Garcia E. (2019). ER and nutrient stress promote assembly of respiratory chain supercomplexes through the PERK-eIF2alpha Axis. *Mol. Cell* 74:e876. 10.1016/j.molcel.2019.03.031 PMC6555668 31023583

[B7] Basso V. Marchesan E. Ziviani E. (2020). A trio has turned into a quartet: DJ-1 interacts with the IP3R-Grp75-VDAC complex to control ER-mitochondria interaction. *Cell Calc.* 87:102186. 10.1016/j.ceca.2020.102186 32120195

[B8] Bergami M. Motori E. (2020). Reweaving the fabric of mitochondrial contact sites in astrocytes. *Front. Cell Dev. Biol.* 8:592651. 10.3389/fcell.2020.592651 33195262 PMC7649784

[B9] Bernard-Marissal N. Chrast R. Schneider B. L. (2018). Endoplasmic reticulum and mitochondria in diseases of motor and sensory neurons: a broken relationship? *Cell Death Dis.* 9:333. 29491369 10.1038/s41419-017-0125-1 PMC5832431

[B10] Bernard-Marissal N. Medard J. J. Azzedine H. Chrast R. (2015). Dysfunction in endoplasmic reticulum-mitochondria crosstalk underlies SIGMAR1 loss of function mediated motor neuron degeneration. *Brain* 138 875–890. 10.1093/brain/awv008 25678561

[B11] Bommiasamy H. Back S. H. Fagone P. Lee K. Meshinchi S. Vink E. (2009). ATF6alpha induces XBP1-independent expansion of the endoplasmic reticulum. *J. Cell Sci.* 122 1626–1636. 10.1242/jcs.045625 19420237 PMC2680102

[B12] Borgmann K. Ghorpade A. (2015). HIV-1, methamphetamine and astrocytes at neuroinflammatory Crossroads. *Front. Microbiol.* 6:1143. 10.3389/fmicb.2015.01143 26579077 PMC4621459

[B13] Borrajo A. Spuch C. Penedo M. A. Olivares J. M. Agis-Balboa R. C. (2021). Important role of microglia in HIV-1 associated neurocognitive disorders and the molecular pathways implicated in its pathogenesis. *Ann. Med.* 53 43–69. 10.1080/07853890.2020.1814962 32841065 PMC7877929

[B14] Brand M. D. Orr A. L. Perevoshchikova I. V. Quinlan C. L. (2013). The role of mitochondrial function and cellular bioenergetics in ageing and disease. *Br. J. Dermatol.* 169 1–8. 10.1111/bjd.12208 23786614 PMC4321783

[B15] Bravo R. Vicencio J. M. Parra V. Troncoso R. Munoz J. P. Bui M. (2011). Increased ER-mitochondrial coupling promotes mitochondrial respiration and bioenergetics during early phases of ER stress. *J. Cell Sci.* 124 2143–2152. 10.1242/jcs.080762 21628424 PMC3113668

[B16] Bravo-Sagua R. Lopez-Crisosto C. Parra V. Rodriguez-Pena M. Rothermel B. A. Quest A. F. (2016). mTORC1 inhibitor rapamycin and ER stressor tunicamycin induce differential patterns of ER-mitochondria coupling. *Sci. Rep.* 6:36394. 27808250 10.1038/srep36394 PMC5093439

[B17] Bronner D. N. Abuaita B. H. Chen X. Fitzgerald K. A. Nunez G. He Y. (2015). Endoplasmic reticulum stress activates the inflammasome via NLRP3- and Caspase-2-driven mitochondrial damage. *Immunity* 43 451–462. 26341399 10.1016/j.immuni.2015.08.008 PMC4582788

[B18] Brown L. A. Scarola J. Smith A. J. Sanberg P. R. Tan J. Giunta B. (2014). The role of tau protein in HIV-associated neurocognitive disorders. *Mol. Neurodegener* 9:40. 25304757 10.1186/1750-1326-9-40 PMC4210623

[B19] Burkewitz K. Feng G. Dutta S. Kelley C. A. Steinbaugh M. Cram E. J. (2020). Atf-6 Regulates Lifespan through ER-mitochondrial calcium homeostasis. *Cell Rep.* 32:108125. 32905769 10.1016/j.celrep.2020.108125 PMC8030272

[B20] Cali T. Ottolini D. Negro A. Brini M. (2012). alpha-Synuclein controls mitochondrial calcium homeostasis by enhancing endoplasmic reticulum-mitochondria interactions. *J. Biol. Chem.* 287 17914–17929. 10.1074/jbc.m111.302794 22453917 PMC3365710

[B21] Cali T. Ottolini D. Negro A. Brini M. (2013). Enhanced parkin levels favor ER-mitochondria crosstalk and guarantee Ca(2+) transfer to sustain cell bioenergetics. *Biochim. Biophys. Acta* 1832 495–508. 10.1016/j.bbadis.2013.01.004 23313576

[B22] Campestrini J. Silveira D. B. Pinto A. R. (2018). HIV-1 Tat-induced bystander apoptosis in Jurkat cells involves unfolded protein responses. *Cell Biochem. Funct.* 36 377–386. 10.1002/cbf.3357 30246458

[B23] Carreras-Sureda A. Jana F. Urra H. Durand S. Mortenson D. E. Sagredo A. (2019). Non-canonical function of IRE1alpha determines mitochondria-associated endoplasmic reticulum composition to control calcium transfer and bioenergetics. *Nat. Cell Biol.* 21 755–767. 10.1038/s41556-019-0329-y 31110288 PMC7246037

[B24] Cieri D. Vicario M. Vallese F. D’orsi B. Berto P. Grinzato A. (2018). Tau localises within mitochondrial sub-compartments and its caspase cleavage affects ER-mitochondria interactions and cellular Ca(2+) handling. *Biochim. Biophys. Acta Mol. Basis Dis.* 1864 3247–3256. 10.1016/j.bbadis.2018.07.011 30006151

[B25] Ciron C. Zheng L. Bobela W. Knott G. W. Leone T. C. Kelly D. P. (2015). PGC-1alpha activity in nigral dopamine neurons determines vulnerability to alpha-synuclein. *Acta Neuropathol. Commun.* 3:16. 25853296 10.1186/s40478-015-0200-8 PMC4379693

[B26] Cisneros I. E. Ghorpade A. (2012). HIV-1, methamphetamine and astrocyte glutamate regulation: combined excitotoxic implications for neuro-AIDS. *Curr. HIV Res.* 10 392–406. 10.2174/157016212802138832 22591363 PMC3580828

[B27] Cody S. L. Vance D. E. (2016). The neurobiology of HIV and its impact on cognitive reserve: A review of cognitive interventions for an aging population. *Neurobiol. Dis.* 92 144–156. 10.1016/j.nbd.2016.01.011 26776767

[B28] Cowan K. Anichtchik O. Luo S. (2019). Mitochondrial integrity in neurodegeneration. *CNS Neurosci. Ther.* 25 825–836. 10.1111/cns.13105 30746905 PMC6566061

[B29] Darbinian N. Darbinyan A. Merabova N. Selzer M. E. Amini S. (2020). HIV-1 and HIV-1-Tat Induce Mitochondrial DNA damage in human neurons. *J. HIV Aids* 6:176. 33506104 10.16966/2380-5536.176 PMC7837619

[B30] de Brito O. M. Scorrano L. (2008). Mitofusin 2 tethers endoplasmic reticulum to mitochondria. *Nature* 456 605–610. 10.1038/nature07534 19052620

[B31] De Vos K. J. Morotz G. M. Stoica R. Tudor E. L. Lau K. F. Ackerley S. (2012). VAPB interacts with the mitochondrial protein PTPIP51 to regulate calcium homeostasis. *Hum. Mol. Genet.* 21 1299–1311. 10.1093/hmg/ddr559 22131369 PMC3284118

[B32] Fan Y. He J. J. (2016). HIV-1 Tat induces unfolded protein response and endoplasmic reticulum stress in astrocytes and causes neurotoxicity through glial fibrillary acidic protein (GFAP) Activation and Aggregation. *J. Biol. Chem.* 291 22819–22829. 10.1074/jbc.m116.731828 27609520 PMC5077214

[B33] Fatima M. Prajapati B. Saleem K. Kumari R. Mohindar Singh Singal C. Seth P. (2017). Novel insights into role of miR-320a-VDAC1 axis in astrocyte-mediated neuronal damage in neuroAIDS. *Glia* 65 250–263. 10.1002/glia.23089 27761954

[B34] Fields J. A. Ellis R. J. (2019). HIV in the cART era and the mitochondrial: immune interface in the CNS. *Int. Rev. Neurobiol.* 145 29–65. 10.1016/bs.irn.2019.04.003 31208526

[B35] Fields J. A. Serger E. Campos S. Divakaruni A. S. Kim C. Smith K. (2016). HIV alters neuronal mitochondrial fission/fusion in the brain during HIV-associated neurocognitive disorders. *Neurobiol. Dis.* 86 154–169. 10.1016/j.nbd.2015.11.015 26611103 PMC4713337

[B36] Filadi R. Greotti E. Turacchio G. Luini A. Pozzan T. Pizzo P. (2015). Mitofusin 2 ablation increases endoplasmic reticulum-mitochondria coupling. *Proc. Natl. Acad. Sci USA* 112 E2174–E2181. 25870285 10.1073/pnas.1504880112 PMC4418914

[B37] Filadi R. Greotti E. Turacchio G. Luini A. Pozzan T. Pizzo P. (2016). Presenilin 2 modulates endoplasmic reticulum-mitochondria coupling by tuning the antagonistic effect of mitofusin 2. *Cell Rep.* 15 2226–2238. 10.1016/j.celrep.2016.05.013 27239030

[B38] Friedman J. R. Lackner L. L. West M. Dibenedetto J. R. Nunnari J. Voeltz G. K. (2011). ER tubules mark sites of mitochondrial division. *Science* 334 358–362. 10.1126/science.1207385 21885730 PMC3366560

[B39] Friedman J. R. Nunnari J. (2014). Mitochondrial form and function. *Nature* 505 335–343. 10.1038/nature12985 24429632 PMC4075653

[B40] Galla L. Redolfi N. Pozzan T. Pizzo P. Greotti E. (2020). Intracellular calcium dysregulation by the Alzheimer’s Disease-linked protein presenilin 2. *Int. J. Mol. Sci.* 21:770. 10.3390/ijms21030770 31991578 PMC7037278

[B41] Garrido-Maraver J. Loh S. H. Y. Martins L. M. (2020). Forcing contacts between mitochondria and the endoplasmic reticulum extends lifespan in a Drosophila model of Alzheimer’s disease. *Biol. Open* 9:bio047530. 31822473 10.1242/bio.047530 PMC6994956

[B42] Gautier C. A. Erpapazoglou Z. Mouton-Liger F. Muriel M. P. Cormier F. Bigou S. (2016). The endoplasmic reticulum-mitochondria interface is perturbed in PARK2 knockout mice and patients with PARK2 mutations. *Hum. Mol. Genet.* 25 2972–2984. 27206984 10.1093/hmg/ddw148

[B43] Gbel J. Engelhardt E. Pelzer P. Sakthivelu V. Jahn H. M. Jevtic M. (2020). Mitochondria-endoplasmic reticulum contacts in reactive astrocytes promote vascular remodeling. *Cell Metab.* 31:e798. 10.1016/j.cmet.2020.03.005 PMC7139200 32220306

[B44] Gelmetti V. De Rosa P. Torosantucci L. Marini E. S. Romagnoli A. Di Rienzo M. (2017). PINK1 and BECN1 relocalize at mitochondria-associated membranes during mitophagy and promote ER-mitochondria tethering and autophagosome formation. *Autophagy* 13 654–669. 10.1080/15548627.2016.1277309 28368777 PMC5388214

[B45] Giorgi C. Marchi S. Pinton P. (2018). The machineries, regulation and cellular functions of mitochondrial calcium. *Nat. Rev. Mol. Cell Biol.* 19 713–730. 10.1038/s41580-018-0052-8 30143745

[B46] Gkogkas C. Middleton S. Kremer A. M. Wardrope C. Hannah M. Gillingwater T. H. (2008). VAPB interacts with and modulates the activity of ATF6. *Hum. Mol. Genet.* 17 1517–1526. 10.1093/hmg/ddn040 18263603

[B47] Gomez-Suaga P. Bravo-San Pedro J. M. Gonzalez-Polo R. A. Fuentes J. M. Niso-Santano M. (2018). ER-mitochondria signaling in Parkinson’s disease. *Cell Death Dis.* 9:337. 29497039 10.1038/s41419-017-0079-3 PMC5832754

[B48] Gomez-Suaga P. Paillusson S. Stoica R. Noble W. Hanger D. P. Miller C. C. J. (2017). The ER-Mitochondria Tethering Complex VAPB-PTPIP51 regulates autophagy. *Curr. Biol.* 27 371–385. 10.1016/j.cub.2016.12.038 28132811 PMC5300905

[B49] Gomez-Suaga P. Perez-Nievas B. G. Glennon E. B. Lau D. H. W. Paillusson S. Morotz G. M. (2019). The VAPB-PTPIP51 endoplasmic reticulum-mitochondria tethering proteins are present in neuronal synapses and regulate synaptic activity. *Acta Neuropathol. Commun.* 7:35. 30841933 10.1186/s40478-019-0688-4 PMC6402140

[B50] Grimm A. Eckert A. (2017). Brain aging and neurodegeneration: from a mitochondrial point of view. *J. Neurochem.* 143 418–431. 10.1111/jnc.14037 28397282 PMC5724505

[B51] Guardia-Laguarta C. Area-Gomez E. Rub C. Liu Y. Magrane J. Becker D. (2014). alpha-Synuclein is localized to mitochondria-associated ER membranes. *J. Neurosci.* 34 249–259. 10.1523/jneurosci.2507-13.2014 24381286 PMC3866487

[B52] Halliday M. Mallucci G. R. (2014). Targeting the unfolded protein response in neurodegeneration: A new approach to therapy. *Neuropharmacology* 76 169–174. 10.1016/j.neuropharm.2013.08.034 24035917

[B53] Hategan A. Masliah E. Nath A. (2019). HIV and Alzheimer’s disease: complex interactions of HIV-Tat with amyloid beta peptide and Tau protein. *J. Neurovirol.* 25 648–660. 10.1007/s13365-019-00736-z 31016584 PMC9056081

[B54] Haughey N. J. Mattson M. P. (2002). Calcium dysregulation and neuronal apoptosis by the HIV-1 proteins Tat and gp120. *J. Acquir. Immune. Defic. Syndr.* 31 S55–S61. 12394783 10.1097/00126334-200210012-00005

[B55] Hayashi T. Su T. P. (2007). Sigma-1 receptor chaperones at the ER-mitochondrion interface regulate Ca(2+) signaling and cell survival. *Cell* 131 596–610. 10.1016/j.cell.2007.08.036 17981125

[B56] He X. Yang W. Zeng Z. Wei Y. Gao J. Zhang B. (2020). NLRP3-dependent pyroptosis is required for HIV-1 gp120-induced neuropathology. *Cell Mol. Immunol.* 17 283–299. 31320730 10.1038/s41423-019-0260-y PMC7052202

[B57] Heaton R. K. Franklin D. R. Ellis R. J. Mccutchan J. A. Letendre S. L. Leblanc S. (2011). HIV-associated neurocognitive disorders before and during the era of combination antiretroviral therapy: differences in rates, nature, and predictors. *J. Neurovirol.* 17 3–16. 21174240 10.1007/s13365-010-0006-1 PMC3032197

[B58] Hedskog L. Pinho C. M. Filadi R. Ronnback A. Hertwig L. Wiehager B. (2013). Modulation of the endoplasmic reticulum-mitochondria interface in Alzheimer’s disease and related models. *Proc. Natl. Acad. Sci. USA* 110 7916–7921. 10.1073/pnas.1300677110 23620518 PMC3651455

[B59] Herrera-Cruz M. S. Simmen T. (2017). Over Six decades of discovery and characterization of the architecture at mitochondria-associated membranes (MAMs). *Adv. Exp. Med. Biol.* 997 13–31. 10.1007/978-981-10-4567-7_2 28815519

[B60] Honrath B. Metz I. Bendridi N. Rieusset J. Culmsee C. Dolga A. M. (2017). Glucose-regulated protein 75 determines ER-mitochondrial coupling and sensitivity to oxidative stress in neuronal cells. *Cell Death Discov.* 3:17076. 29367884 10.1038/cddiscovery.2017.76 PMC5672593

[B61] Hu X. T. (2016). HIV-1 tat-mediated calcium dysregulation and neuronal dysfunction in vulnerable brain regions. *Curr. Drug Targets* 17 4–14. 10.2174/1389450116666150531162212 26028040 PMC4772427

[B62] Huang C. Y. Chiang S. F. Lin T. Y. Chiou S. H. Chow K. C. (2012). HIV-1 Vpr triggers mitochondrial destruction by impairing Mfn2-mediated ER-mitochondria interaction. *PLoS One* 7:e33657. 10.1371/journal.pone.0033657 22438978 PMC3306277

[B63] Iwasawa R. Mahul-Mellier A. L. Datler C. Pazarentzos E. Grimm S. (2011). Fis1 and Bap31 bridge the mitochondria-ER interface to establish a platform for apoptosis induction. *EMBO J.* 30 556–568. 10.1038/emboj.2010.346 21183955 PMC3034017

[B64] Janikiewicz J. Szymanski J. Malinska D. Patalas-Krawczyk P. Michalska B. Duszynski J. (2018). Mitochondria-associated membranes in aging and senescence: structure, function, and dynamics. *Cell Death Dis.* 9:332. 29491385 10.1038/s41419-017-0105-5 PMC5832430

[B65] Jones G. J. Barsby N. L. Cohen E. A. Holden J. Harris K. Dickie P. (2007). HIV-1 Vpr causes neuronal apoptosis and in vivo neurodegeneration. *J. Neurosci.* 27 3703–3711. 10.1523/jneurosci.5522-06.2007 17409234 PMC6672409

[B66] Khan N. Haughey N. J. Nath A. Geiger J. D. (2019). Involvement of organelles and inter-organellar signaling in the pathogenesis of HIV-1 associated neurocognitive disorder and Alzheimer’s disease. *Brain Res.* 1722:146389. 31425679 10.1016/j.brainres.2019.146389 PMC6755067

[B67] Kim J. Yoon J. H. Kim Y. S. (2013). HIV-1 Tat interacts with and regulates the localization and processing of amyloid precursor protein. *PLoS One* 8:e77972. 10.1371/journal.pone.0077972 24312169 PMC3843664

[B68] Kruman II. Nath A. Mattson M. P. (1998). HIV-1 protein Tat induces apoptosis of hippocampal neurons by a mechanism involving caspase activation, calcium overload, and oxidative stress. *Exp. Neurol.* 154 276–288. 10.1006/exnr.1998.6958 9878167

[B69] Lau D. H. W. Hartopp N. Welsh N. J. Mueller S. Glennon E. B. Morotz G. M. (2018). Disruption of ER-mitochondria signalling in fronto-temporal dementia and related amyotrophic lateral sclerosis. *Cell Death Dis.* 9:327. 29491392 10.1038/s41419-017-0022-7 PMC5832427

[B70] Lau D. H. W. Paillusson S. Hartopp N. Rupawala H. Morotz G. M. Gomez-Suaga P. (2020). Disruption of endoplasmic reticulum-mitochondria tethering proteins in post-mortem Alzheimer’s disease brain. *Neurobiol. Dis.* 143:105020. 10.1016/j.nbd.2020.105020 32682953 PMC7794060

[B71] Leal N. S. Dentoni G. Schreiner B. Naia L. Piras A. Graff C. (2020). Amyloid Beta-Peptide increases mitochondria-endoplasmic reticulum contact altering mitochondrial function and autophagosome formation in Alzheimer’s Disease-Related Models. *Cells* 9:2552. 10.3390/cells9122552 33260715 PMC7760163

[B72] Leal N. S. Martins L. M. (2021). Mind the Gap: mitochondria and the endoplasmic reticulum in neurodegenerative diseases. *Biomedicines* 9:227. 10.3390/biomedicines9020227 33672391 PMC7926795

[B73] Leal N. S. Schreiner B. Pinho C. M. Filadi R. Wiehager B. Karlstrom H. (2016). Mitofusin-2 knockdown increases ER-mitochondria contact and decreases amyloid beta-peptide production. *J. Cell Mol. Med.* 20 1686–1695. 10.1111/jcmm.12863 27203684 PMC4988279

[B74] Lebeau J. Saunders J. M. Moraes V. W. R. Madhavan A. Madrazo N. Anthony M. C. (2018). The PERK Arm of the Unfolded protein response regulates mitochondrial morphology during acute endoplasmic reticulum stress. *Cell Rep.* 22 2827–2836. 10.1016/j.celrep.2018.02.055 29539413 PMC5870888

[B75] Liao Y. Dong Y. Cheng J. (2017). The function of the mitochondrial calcium uniporter in neurodegenerative disorders. *Int. J. Mol. Sci.* 18:248. 10.3390/ijms18020248 28208618 PMC5343785

[B76] Liao Y. Dong Y. Cheng J. (2020). The molecular determinants of mitochondrial membrane contact with er, lysosomes and peroxisomes in neuronal physiology and pathology. *Front. Cell Neurosci.* 14:194. 10.3389/fncel.2020.00194 32848610 PMC7427582

[B77] Lindl K. A. Akay C. Wang Y. White M. G. Jordan-Sciutto K. L. (2007). Expression of the endoplasmic reticulum stress response marker, BiP, in the central nervous system of HIV-positive individuals. *Neuropathol. Appl. Neurobiol.* 33 658–669. 10.1111/j.1365-2990.2007.00866.x 17931354

[B78] Liu Y. Ma X. Fujioka H. Liu J. Chen S. Zhu X. (2019). DJ-1 regulates the integrity and function of ER-mitochondria association through interaction with IP3R3-Grp75-VDAC1. *Proc. Natl. Acad. Sci. USA* 116 25322–25328. 10.1073/pnas.1906565116 31767755 PMC6911199

[B79] Ma R. Yang L. Niu F. Buch S. (2016). HIV Tat-mediated induction of human brain microvascular endothelial cell apoptosis involves endoplasmic reticulum stress and mitochondrial dysfunction. *Mol. Neurobiol.* 53 132–142. 10.1007/s12035-014-8991-3 25409632 PMC4787264

[B80] Mackiewicz M. M. Overk C. Achim C. L. Masliah E. (2019). Pathogenesis of age-related HIV neurodegeneration. *J. Neurovirol.* 25 622–633. 10.1007/s13365-019-00728-z 30790184 PMC6703984

[B81] Malli R. Graier W. F. (2019). IRE1alpha modulates ER and mitochondria crosstalk. *Nat. Cell Biol.* 21 667–668. 10.1038/s41556-019-0338-x 31110289

[B82] Manfredi G. Kawamata H. (2016). Mitochondria and endoplasmic reticulum crosstalk in amyotrophic lateral sclerosis. *Neurobiol. Dis.* 90 35–42. 10.1016/j.nbd.2015.08.004 26282323 PMC4754163

[B83] Manninen A. Saksela K. (2002). HIV-1 Nef interacts with inositol trisphosphate receptor to activate calcium signaling in T cells. *J. Exp. Med.* 195 1023–1032. 10.1084/jem.20012039 11956293 PMC2193699

[B84] Marchi S. Bittremieux M. Missiroli S. Morganti C. Patergnani S. Sbano L. (2017). Endoplasmic reticulum-mitochondria communication through Ca(2+) Signaling: the importance of mitochondria-associated membranes (MAMs). *Adv. Exp. Med. Biol.* 997 49–67. 10.1007/978-981-10-4567-7_4 28815521

[B85] Martinez A. Lopez N. Gonzalez C. Hetz C. (2019). Targeting of the unfolded protein response (UPR) as therapy for Parkinson’s disease. *Biol. Cell* 111 161–168. 10.1111/boc.201800068 30860281

[B86] Meeker R. B. Poulton W. Clary G. Schriver M. Longo F. M. (2016). Novel p75 neurotrophin receptor ligand stabilizes neuronal calcium, preserves mitochondrial movement and protects against HIV associated neuropathogenesis. *Exp. Neurol.* 275 182–198. 10.1016/j.expneurol.2015.09.012 26424436 PMC4688079

[B87] Misko A. Jiang S. Wegorzewska I. Milbrandt J. Baloh R. H. (2010). Mitofusin 2 is necessary for transport of axonal mitochondria and interacts with the Miro/Milton complex. *J. Neurosci.* 30 4232–4240. 10.1523/jneurosci.6248-09.2010 20335458 PMC2852190

[B88] Misko A. L. Sasaki Y. Tuck E. Milbrandt J. Baloh R. H. (2012). Mitofusin2 mutations disrupt axonal mitochondrial positioning and promote axon degeneration. *J. Neurosci.* 32 4145–4155. 22442078 10.1523/JNEUROSCI.6338-11.2012 PMC3319368

[B89] Modi S. Lopez-Domenech G. Halff E. F. Covill-Cooke C. Ivankovic D. Melandri D. (2019). Miro clusters regulate ER-mitochondria contact sites and link cristae organization to the mitochondrial transport machinery. *Nat. Commun.* 10:4399. 31562315 10.1038/s41467-019-12382-4 PMC6764964

[B90] Moltedo O. Remondelli P. Amodio G. (2019). The mitochondria-endoplasmic reticulum contacts and their critical role in aging and age-associated diseases. *Front. Cell Dev. Biol.* 7:172. 10.3389/fcell.2019.00172 31497601 PMC6712070

[B91] Monaco A. Fraldi A. (2020). Protein aggregation and dysfunction of autophagy-lysosomal pathway: a vicious cycle in lysosomal storage diseases. *Front. Mol. Neurosci.* 13:37. 10.3389/fnmol.2020.00037 32218723 PMC7079699

[B92] Mori T. Hayashi T. Hayashi E. Su T. P. (2013). Sigma-1 receptor chaperone at the ER-mitochondrion interface mediates the mitochondrion-ER-nucleus signaling for cellular survival. *PLoS One* 8:e76941. 10.1371/journal.pone.0076941 24204710 PMC3799859

[B93] Munoz J. P. Ivanova S. Sanchez-Wandelmer J. Martinez-Cristobal P. Noguera E. Sancho A. (2013). Mfn2 modulates the UPR and mitochondrial function via repression of PERK. *EMBO J.* 32 2348–2361. 10.1038/emboj.2013.168 23921556 PMC3770335

[B94] Natarajaseenivasan K. Cotto B. Shanmughapriya S. Lombardi A. A. Datta P. K. Madesh M. (2018). Astrocytic metabolic switch is a novel etiology for Cocaine and HIV-1 Tat-mediated neurotoxicity. *Cell Death Dis.* 9:415. 10.1038/s41419-018-0422-3 29549313 PMC5856787

[B95] Nishimura A. L. Mitne-Neto M. Silva H. C. Richieri-Costa A. Middleton S. Cascio D. (2004). A mutation in the vesicle-trafficking protein VAPB causes late-onset spinal muscular atrophy and amyotrophic lateral sclerosis. *Am. J. Hum. Genet.* 75 822–831. 10.1086/425287 15372378 PMC1182111

[B96] Nooka S. Ghorpade A. (2017). HIV-1-associated inflammation and antiretroviral therapy regulate astrocyte endoplasmic reticulum stress responses. *Cell Death Discov.* 3:17061. 10.1038/cddiscovery.2017.61 29354290 PMC5712632

[B97] Nooka S. Ghorpade A. (2018). Organellar stress intersects the astrocyte endoplasmic reticulum, mitochondria and nucleolus in HIV associated neurodegeneration. *Cell Death Dis.* 9:317. 10.1038/s41419-018-0341-3 29472528 PMC5833429

[B98] Norman J. P. Perry S. W. Reynolds H. M. Kiebala M. De Mesy Bentley K. L. Trejo M. (2008). HIV-1 Tat activates neuronal ryanodine receptors with rapid induction of the unfolded protein response and mitochondrial hyperpolarization. *PLoS One* 3:e3731. 10.1371/journal.pone.0003731 19009018 PMC2579580

[B99] Ojeda D. S. Grasso D. Urquiza J. Till A. Vaccaro M. I. Quarleri J. (2018). Cell death is counteracted by mitophagy in HIV-productively infected astrocytes but is promoted by inflammasome activation among non-productively infected cells. *Front. Immunol.* 9:2633. 10.3389/fimmu.2018.02633 30515154 PMC6255949

[B100] Ottolini D. Cali T. Negro A. Brini M. (2013). The Parkinson disease-related protein DJ-1 counteracts mitochondrial impairment induced by the tumour suppressor protein p53 by enhancing endoplasmic reticulum-mitochondria tethering. *Hum. Mol. Genet.* 22 2152–2168. 10.1093/hmg/ddt068 23418303

[B101] Paillusson S. Gomez-Suaga P. Stoica R. Little D. Gissen P. Devine M. J. (2017). alpha-Synuclein binds to the ER-mitochondria tethering protein VAPB to disrupt Ca(2+) homeostasis and mitochondrial ATP production. *Acta Neuropathol.* 134 129–149. 10.1007/s00401-017-1704-z 28337542 PMC5486644

[B102] Paillusson S. Stoica R. Gomez-Suaga P. Lau D. H. W. Mueller S. Miller T. (2016). There’s something wrong with my MAM; the ER-mitochondria axis and neurodegenerative diseases. *Trends Neurosci.* 39 146–157. 10.1016/j.tins.2016.01.008 26899735 PMC4780428

[B103] Perreault S. Bousquet O. Lauzon M. Paiement J. Leclerc N. (2009). Increased association between rough endoplasmic reticulum membranes and mitochondria in transgenic mice that express P301L tau. *J. Neuropathol. Exp. Neurol.* 68 503–514. 10.1097/NEN.0b013e3181a1fc49 19525898

[B104] Picca A. Calvani R. Coelho-Junior H. J. Landi F. Bernabei R. Marzetti E. (2020). Inter-organelle membrane contact sites and mitochondrial quality control during aging: a geroscience view. *Cells* 9:598. 10.3390/cells9030598 32138154 PMC7140483

[B105] Pinton P. (2018). Mitochondria-associated membranes (MAMs) and pathologies. *Cell Death Dis.* 9:413. 10.1038/s41419-018-0424-1 29549303 PMC5856760

[B106] Popov L. D. (2020). Mitochondrial biogenesis: an update. *J. Cell Mol. Med.* 24 4892–4899. 10.1111/jcmm.15194 32279443 PMC7205802

[B107] Priyanka Wadhwa R. Chaudhuri R. Nag T. C. Seth P. (2020). Novel role of mortalin in attenuating HIV-1 Tat-mediated astrogliosis. *J. Neuroinflammation* 17:276. 10.1186/s12974-020-01912-3 32951595 PMC7504834

[B108] Raeisossadati R. Ferrari M. F. R. (2020). Mitochondria-ER tethering in neurodegenerative diseases. *Cell Mol. Neurobiol.* 2020:9. 10.1007/s10571-020-01008-9 33196974 PMC11441217

[B109] Rainbolt T. K. Saunders J. M. Wiseman R. L. (2014). Stress-responsive regulation of mitochondria through the ER unfolded protein response. *Trends Endocrinol. Metab.* 25 528–537. 10.1016/j.tem.2014.06.007 25048297

[B110] Rawat P. Teodorof-Diedrich C. Spector S. A. (2019). Human immunodeficiency virus Type-1 single-stranded RNA activates the NLRP3 inflammasome and impairs autophagic clearance of damaged mitochondria in human microglia. *Glia* 67 802–824. 10.1002/glia.23568 30582668 PMC6493331

[B111] Remondelli P. Renna M. (2017). The endoplasmic reticulum unfolded protein response in neurodegenerative disorders and its potential therapeutic significance. *Front. Mol. Neurosci.* 10:187. 10.3389/fnmol.2017.00187 28670265 PMC5472670

[B112] Rizzuto R. De Stefani D. Raffaello A. Mammucari C. (2012). Mitochondria as sensors and regulators of calcium signalling. *Nat. Rev. Mol. Cell Biol.* 13 566–578. 10.1038/nrm3412 22850819

[B113] Robertson K. R. Smurzynski M. Parsons T. D. Wu K. Bosch R. J. Wu J. (2007). The prevalence and incidence of neurocognitive impairment in the HAART era. *AIDS* 21 1915–1921. 10.1097/QAD.0b013e32828e4e27 17721099

[B114] Rodriguez-Arribas M. Yakhine-Diop S. M. S. Pedro J. M. B. Gomez-Suaga P. Gomez-Sanchez R. Martinez-Chacon G. (2017). Mitochondria-Associated Membranes (MAMs): overview and its role in parkinson’s disease. *Mol. Neurobiol.* 54 6287–6303. 27714635 10.1007/s12035-016-0140-8

[B115] Rozzi S. J. Avdoshina V. Fields J. A. Mocchetti I. (2018). Human immunodeficiency virus Tat impairs mitochondrial fission in neurons. *Cell Death Discov.* 4:8. 10.1038/s41420-017-0013-6 29531805 PMC5841280

[B116] Saito A. Imaizumi K. (2018). Unfolded protein response-dependent communication and contact among endoplasmic reticulum, mitochondria, and plasma membrane. *Int. J. Mol. Sci.* 19:3215. 10.3390/ijms19103215 30340324 PMC6213962

[B117] Schank M. Zhao J. Moorman J. P. Yao Z. Q. (2021). The Impact of HIV- and ART-induced mitochondrial dysfunction in cellular senescence and aging. *Cells* 10:174. 10.3390/cells10010174 33467074 PMC7830696

[B118] Schreiner B. Hedskog L. Wiehager B. Ankarcrona M. (2015). Amyloid-beta peptides are generated in mitochondria-associated endoplasmic reticulum membranes. *J. Alzheimers Dis.* 43 369–374. 10.3233/JAD-132543 25096627

[B119] Shah A. Vaidya N. K. Bhat H. K. Kumar A. (2016). HIV-1 gp120 induces type-1 programmed cell death through ER stress employing IRE1alpha. JNK and AP-1 pathway. *Sci. Rep.* 6:18929. 10.1038/srep18929 26740125 PMC4703964

[B120] Shelton M. N. Huang M. B. Ali S. A. Powell M. D. Bond V. C. (2012). Secretion modification region-derived peptide disrupts HIV-1 Nef’s interaction with mortalin and blocks virus and Nef exosome release. *J. Virol.* 86 406–419. 10.1128/JVI.05720-11 22013042 PMC3255900

[B121] Shirokova O. M. Pchelin P. V. Mukhina I. V. (2020). MERCs. the novel assistant to neurotransmission? *Front. Neurosci.* 14:589319. 10.3389/fnins.2020.589319 33240039 PMC7680918

[B122] Silva J. G. Martins N. P. Henriques R. Soares H. (2016). HIV-1 Nef Impairs the formation of calcium membrane territories controlling the signaling nanoarchitecture at the immunological synapse. *J. Immunol.* 197 4042–4052. 10.4049/jimmunol.1601132 27798165 PMC5098604

[B123] Simioni S. Cavassini M. Annoni J. M. Rimbault Abraham A. Bourquin I. Schiffer V. (2010). Cognitive dysfunction in HIV patients despite long-standing suppression of viremia. *Aids* 24 1243–1250. 10.1097/QAD.0b013e3283354a7b 19996937

[B124] Simmen T. Aslan J. E. Blagoveshchenskaya A. D. Thomas L. Wan L. Xiang Y. (2005). PACS-2 controls endoplasmic reticulum-mitochondria communication and Bid-mediated apoptosis. *EMBO J.* 24 717–729. 10.1038/sj.emboj.7600559 15692567 PMC549619

[B125] Son S. M. Byun J. Roh S. E. Kim S. J. Mook-Jung I. (2014). Reduced IRE1alpha mediates apoptotic cell death by disrupting calcium homeostasis via the InsP3 receptor. *Cell Death Dis.* 5:e1188. 10.1038/cddis.2014.129 24743743 PMC4001297

[B126] Stoica R. De Vos K. J. Paillusson S. Mueller S. Sancho R. M. Lau K. F. (2014). ER-mitochondria associations are regulated by the VAPB-PTPIP51 interaction and are disrupted by ALS/FTD-associated TDP-43. *Nat. Commun.* 5:3996. 10.1038/ncomms4996 24893131 PMC4046113

[B127] Stoica R. Paillusson S. Gomez-Suaga P. Mitchell J. C. Lau D. H. Gray E. H. (2016). ALS/FTD-associated FUS activates GSK-3beta to disrupt the VAPB-PTPIP51 interaction and ER-mitochondria associations. *EMBO Rep.* 17 1326–1342. 10.15252/embr.201541726 27418313 PMC5007559

[B128] Sweeney P. Park H. Baumann M. Dunlop J. Frydman J. Kopito R. (2017). Protein misfolding in neurodegenerative diseases: implications and strategies. *Transl. Neurodegener.* 6:6. 10.1186/s40035-017-0077-5 28293421 PMC5348787

[B129] Swinton M. K. Carson A. Telese F. Sanchez A. B. Soontornniyomkij B. Rad L. (2019). Mitochondrial biogenesis is altered in HIV+ brains exposed to ART: Implications for therapeutic targeting of astroglia. *Neurobiol. Dis.* 130:104502. 10.1016/j.nbd.2019.104502 31238091 PMC6714553

[B130] Szabo L. Eckert A. Grimm A. (2020). Insights into disease-associated tau impact on mitochondria. *Int. J. Mol. Sci.* 21:6344. 10.3390/ijms21176344 32882957 PMC7503371

[B131] Tambini M. D. Pera M. Kanter E. Yang H. Guardia-Laguarta C. Holtzman D. (2016). ApoE4 upregulates the activity of mitochondria-associated ER membranes. *EMBO Rep.* 17 27–36. 10.15252/embr.201540614 26564908 PMC4718413

[B132] Teodorof-Diedrich C. Spector S. A. (2018). Human immunodeficiency virus type 1 gp120 and tat induce mitochondrial fragmentation and incomplete mitophagy in human neurons. *J. Virol.* 92:e00993 10.1128/JVI.00993-18 30158296 PMC6206485

[B133] Thangaraj A. Periyasamy P. Liao K. Bendi V. S. Callen S. Pendyala G. (2018). HIV-1 TAT-mediated microglial activation: role of mitochondrial dysfunction and defective mitophagy. *Autophagy* 14 1596–1619. 10.1080/15548627.2018.1476810 29966509 PMC6135576

[B134] van Vliet A. R. Agostinis P. (2016). When under pressure, get closer: PERKing up membrane contact sites during ER stress. *Biochem. Soc. Trans.* 44 499–504. 27068961 10.1042/BST20150272

[B135] van Vliet A. R. Agostinis P. (2018). Mitochondria-associated membranes and ER Stress. *Curr. Top Microbiol. Immunol.* 414 73–102. 28349285 10.1007/82_2017_2

[B136] van Vliet A. R. Verfaillie T. Agostinis P. (2014). New functions of mitochondria associated membranes in cellular signaling. *Biochim. Biophys. Acta* 1843 2253–2262. 24642268 10.1016/j.bbamcr.2014.03.009

[B137] Verfaillie T. Rubio N. Garg A. D. Bultynck G. Rizzuto R. Decuypere J. P. (2012). PERK is required at the ER-mitochondrial contact sites to convey apoptosis after ROS-based ER stress. *Cell Death Differ.* 19 1880– 1891. 22705852 10.1038/cdd.2012.74 PMC3469056

[B138] Vincenz-Donnelly L. Hipp M. S. (2017). The endoplasmic reticulum: A hub of protein quality control in health and disease. *Free Radic. Biol. Med.* 108 383–393. 28363604 10.1016/j.freeradbiomed.2017.03.031

[B139] Wang Y. Santerre M. Tempera I. Martin K. Mukerjee R. Sawaya B. E. (2017). HIV-1 Vpr disrupts mitochondria axonal transport and accelerates neuronal aging. *Neuropharmacology* 117 364–375. 10.1016/j.neuropharm.2017.02.008 28212984 PMC5397298

[B140] Weng T. Y. Tsai S. A. Su T. P. (2017). Roles of sigma-1 receptors on mitochondrial functions relevant to neurodegenerative diseases. *J. Biomed. Sci.* 24:74. 10.1186/s12929-017-0380-6 28917260 PMC5603014

[B141] Wilson E. L. Metzakopian E. (2020). ER-mitochondria contact sites in neurodegeneration: genetic screening approaches to investigate novel disease mechanisms. *Cell Death Differ.* 2020:723. 10.1038/s41418-020-00723-6 33335290 PMC8185109

[B142] Yang M. Li C. Yang S. Xiao Y. Xiong X. Chen W. (2020). Mitochondria-Associated ER membranes - the origin site of autophagy. *Front. Cell Dev. Biol.* 8:595. 10.3389/fcell.2020.00595 32766245 PMC7378804

[B143] Yoboue E. D. Sitia R. Simmen T. (2018). Redox crosstalk at endoplasmic reticulum (ER) membrane contact sites (MCS) uses toxic waste to deliver messages. *Cell Death Dis.* 9:331. 10.1038/s41419-017-0033-4 29491367 PMC5832433

[B144] Zampese E. Fasolato C. Kipanyula M. J. Bortolozzi M. Pozzan T. Pizzo P. (2011). Presenilin 2 modulates endoplasmic reticulum (ER)-mitochondria interactions and Ca2+ cross-talk. *Proc. Natl. Acad. Sci. USA* 108 2777–2782. 10.1073/pnas.1100735108 21285369 PMC3041131

